# Review of Antimicrobial Properties of Titanium Dioxide Nanoparticles

**DOI:** 10.3390/ijms251910519

**Published:** 2024-09-29

**Authors:** Dmitriy A. Serov, Ann V. Gritsaeva, Fatikh M. Yanbaev, Alexander V. Simakin, Sergey V. Gudkov

**Affiliations:** 1 Prokhorov General Physics Institute of the Russian Academy of Sciences, Vavilove St. 38, 119991 Moscow, Russia; dmitriy_serov_91@mail.ru (D.A.S.); anngritsaeva@mail.ru (A.V.G.); s_makariy@rambler.ru (S.V.G.); 2 Federal Research Center Kazan Scientific Center of Russian Academy of Sciences, Lobachevskogo St. 2/31, Tatarstan, 420111 Kazan, Russia; f.yanbayev@knc.ru; 3 Institute of Biology and Biomedicine, Lobachevsky State University of Nizhny Novgorod Institute, Gagarin Av. 23, 603105 Nizhny Novgorod, Russia

**Keywords:** nanoparticles, TiO_2_, nano-titania, titanium oxide, antibacterial activity, antifungal activity, photocatalysis

## Abstract

There is a growing interest in the utilization of metal oxide nanoparticles as antimicrobial agents. This review will focus on titanium dioxide nanoparticles (TiO_2_ NPs), which have been demonstrated to exhibit high antimicrobial activity against bacteria and fungi, chemical stability, low toxicity to eukaryotic cells, and therefore high biocompatibility. Despite the extensive research conducted in this field, there is currently no consensus on how to enhance the antimicrobial efficacy of TiO_2_ NPs. The aim of this review is to evaluate the influence of various factors, including particle size, shape, composition, and synthesis parameters, as well as microbial type, on the antibacterial activity of TiO_2_ NPs against bacteria and fungi. Furthermore, the review offers a comprehensive overview of the methodologies employed in the synthesis and characterization of TiO_2_ NPs. The antimicrobial activity of TiO_2_ exhibits a weak dependence on the microorganism species. A tendency towards increased antibacterial activity is observed with decreasing TiO_2_ NP size. The dependence on the shape and composition is more pronounced. The most pronounced antimicrobial potential is exhibited by amorphous NPs and NPs doped with inorganic compounds. This review may be of interest to specialists in biology, medicine, chemistry, and other related fields.

## 1. Introduction

Resistance of microorganisms to antimicrobial drugs is one of the key global health problems [1]. Antibiotic resistance was known at the dawn of the “antibiotic era” since the discovery of penicillin. Already 10 years after the introduction of penicillin into medical practice, it turned out to be largely ineffective [2]. A temporary solution to this problem was the synthesis of new classes of antibiotics; however, new resistant strains soon emerged [3,4,5]. Bacteremia caused by antibiotic-resistant strains is the reason for over 1.2 million illnesses worldwide, 23,000 deaths annually in the USA alone and over 25,000 in Europe [6]. Among the antibiotic-resistant strains with the greatest epidemiological significance, it is worth noting the methicillin-resistant bacteria *Staphylococcus aureus* [7,8,9], vancomycin-resistant *Enterococcus faecium* and *Enterococcus faecalis*, drug-resistant *Streptococcus pneumoniae* [10,11], drug-resistant *Streptococcus pneumonia* [12,13,14,15], drug-resistant *Mycobacterium Tuberculosis* [16,17], etc. Among the bacteria *Streptococcus pneumonia* in patient isolates in Japan, up to 80% of strains are penicillin-resistant [13]. Today, there is an active search for a solution to this problem, as evidenced by the continuing increase in the number of publications and citations on “antibacterial resistant bacteria” [18]. Unfortunately, a single effective solution to the given problems has been discovered. The five mechanisms of bacterial antimicrobial resistance have been described: enzymatic degradation of antibiotics (for example, by bacterial β-lactamases); modification of the antibiotic target, i.e., the target becomes modified so that the antibiotic is no longer able to bind to its site of action; control of drug entry through mutations in bacterial cell wall porin molecules and membrane modifications; activation of efflux pump systems that are able to pump antibiotics out of the cell before interactions of antibiotic with a target [19]. Possible paths to overcoming microbial resistance include the use of antibiotic adjuvants (inhibitors of β-lactamase, inhibitors of efflux pumps, and outer membrane permeabilizers, antivirulence compounds), bacteriolytic enzymes, metal and metal oxide nanoparticles [20,21,22,23]. It is now suggested that the problem of microbial resistance is not a private problem of hospitals and human homes. A concept is being developed according to which microbial resistance affects not only humans but also animals and the environment. This concept received the title “The interdisciplinary One Health (OH) approach” [24]. If we accept this doctrine, it becomes obvious that methods for overcoming microbial resistance should find easy application not only in medicine but in other areas of economic activity, in particular, agriculture.

A potential way to overcome microbial resistance is the application of metal and metal oxide nanoparticles [22,23]. Nanotechnology and nanobiotechnology are currently at a stage of rapid development and are already successfully applied in a variety of fields: agriculture and the food industry, wastewater treatment, environmental monitoring, biology and medicine, cancer therapy, and targeted drug delivery systems [23,25,26,27,28,29,30,31,32,33]. The market for nanotechnology is experiencing consistent growth on an annual basis. In 2016, the production of nanoproducts reached a value of $12.7 billion, representing an annual growth rate of approximately 9.5%. In 2016, the contribution of nanotechnology products to the global gross domestic product (GDP) was 0.01%. As forecast by Rosnanotech, the percentage of nanoproducts in GDP is expected to reach 0.5% by 2018, 2% by 2020, and 40% by 2035 [34]. The market value of these products is estimated to be between $4.1 billion and $14.7 billion, depending on the evaluation criteria [35].

**Figure 1:**
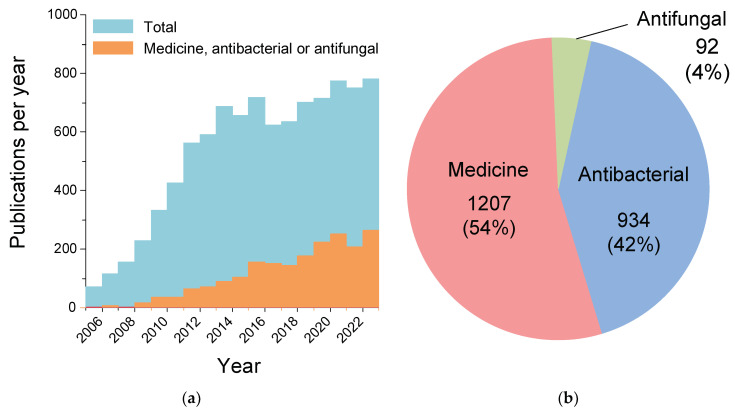
PubMed database information (https://pubmed.ncbi.nlm.nih.gov/ accessed on 27 August 2024): (**a**) The dynamics of the number of publications according to the search by keywords «titanium oxide nanoparticles» (blue color, group I) and «titanium oxide nanoparticles + [«antibacterial» or «antifungal» or «medicine»]» (orange, group II); (**b**) the shares of publications in individual categories in group II for the entire analyzed time interval (2005–2023).

Titanium (Ti) is a chemical element in group 4 of period 4 of the periodic table of chemical elements, with atomic number 22 and a molar mass of 47.867 g/mol [36]. It is classified as a transition metal. Ti is capable of forming a wide range of oxides with different O/Ti stoichiometry: Ti_3_O, Ti_2_O_2_, Ti_6_O, Ti_5_O_9_, Ti_4_O_7_, TiO, TiO_2,_ and Ti_2_O and their combinations [37]. Non-stoichiometric titanium oxides or titanium suboxides (TiO_2−x_ and TinO_2n−1_) have increased electrical conductivity and visible light absorptivity [38]. Due to their unique properties, NPs and nanostructures based on non-stoichiometric titanium oxides can find applications in the development of new generation batteries and solar panels, anti-corrosion coatings, optoelectric devices, volatile organic compound gas sensors, wastewater treatment devices, etc. [37,38,39]. TiO_2_ is usually represented in NPs when studying antibacterial properties. Titanium dioxide (TiO_2_) has several crystalline forms. An anatase, brookite, and rutile are the three most common [40]. All three crystal structures are composed of TiO_6_ octahedra; however, their spatial arrangement differs. Rutile has a tetragonal structure in which the two opposite edges of each octahedron are separated to form linear chains along the direction. The TiO_6_ chains are then joined to each other via a corner bond. Anatase does not share corners but shares four edges per octahedron. The crystal structure of anatase can be viewed as zigzag chains of octahedra linked together by their shared edges. In the crystal lattice of brookite, the octahedra share three edges as well as corners, and the dominant structural feature is the edge-sharing chain, the TiO_6_ octahedra being parallel to the c-axis and cross-linked by the shared edges. The crystal structure and some physical properties of brookite appear to be between those of anatase and rutile. Titanium, its compounds, and Ti-containing alloys find numerous applications in a wide variety of areas of human economic activity: aerospace, mechanical engineering, chemical, automotive, agricultural, food industries, electronics, and jewelry production [41,42,43,44]. Titanium alloys are used in medicine (prosthetics) due to their inertness and high biocompatibility with human tissues [45,46]. Antibacterial and antifungal properties have been found in titanium dioxide nanoparticles TiO_2_ [47,48,49]. According to the PubMed database alone, the antimicrobial properties of TiO_2_ NPs have been described in >1000 articles since 2000 (Figure 1a). It is noteworthy that the share of papers devoted to medical applications and antimicrobial activity of TiO_2_ NPs increased from 4% to 39% of the total number of publications devoted to TiO_2_ NPs over the period 2005–2023, and the share of publications with the keywords “antibacterial” or “antifungal” increased from 2% to 18%. These statistics indicate the high potential of TiO_2_ NPs as antimicrobial agents. The share of papers devoted to the study of the antibacterial properties of TiO_2_ NPs is 10 times higher than the number of papers studying the antifungal properties of TiO_2_ NPs (Figure 1b). This may be due to the methodological features of the study of antifungal effects. The high inertness of TiO_2_ NPs makes it a more attractive candidate for the creation of antimicrobial NPs compared to a number of other metals and their oxides, such as ZnO or iron oxides [50,51].

A large amount of accumulated literature data requires systematization and generalized quantitative analysis to identify more promising directions of work and recommendations for future studies. Recently, several detailed reviews on the antimicrobial effects of TiO_2_ NPs have been published [52]. These reviews describe in detail the structural features of TiO_2_ NPs, methods of synthesis and modification, and attempt to quantify the dependence of the antimicrobial activity of TiO_2_ NPs. on their morphology and surface modification [52,53]. However, at the moment, there is still no unified picture of what factors determine the magnitude of antimicrobial activity of TiO_2_ NPs. In this review, we have attempted to summarize the literature data on the antimicrobial properties of TiO_2_ NPs in order to identify new dependencies of antimicrobial potential on the systematic affiliation of microorganisms, the morphology of the NPs (size and shape), the method of synthesis and the method of surface modification/composition of TiO_2_ NPs. In addition, approaches and methods of synthesis of TiO_2_ NPs, as well as methods of their investigation and characterization, will be briefly described.

## 2. TiO_2_ NPs Synthesis Methods

**Figure 2:**
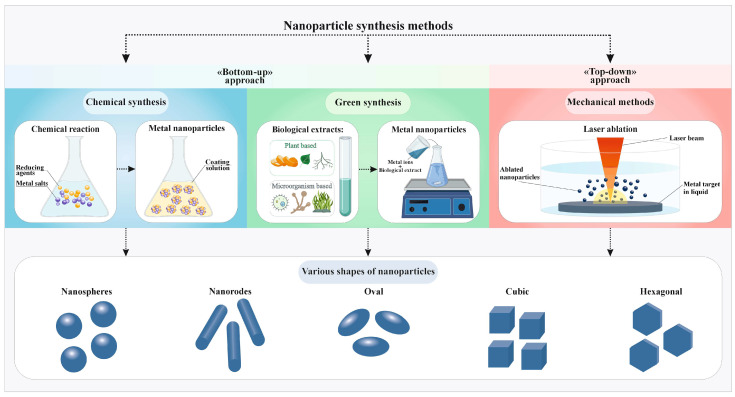
Methods for the synthesis of TiO_2_ NPs (references in the text).

The methods of synthesis of TiO_2_ NPs are quite diverse and include methods and their modifications. All synthesis methods can be divided into two large groups according to the “Bottom-up” and “Top-down” approach (Figure 2).

The first approach is based on the formation of NPs by aggregation of individual molecules into a particle, or, in other words, the transformation of precursor solutions into NP colloids. This group includes chemical synthesis methods: sol−gel method, hydrolysis of precursors, for example, TiCl_4_, electrochemical method, sonochemical (ultrasonic) method, hydrothermal method, photocatalysis (proton-induced), microwave method, co-precipitation of a mixture of salts [54,55,56,57,58,59,60,61]. Halogenates, hydroxides, or organic compounds of titanium are used as precursors for chemical synthesis, for example, titanium tetrabutylate Ti(OBu)_4_ titanium oxy sulfate or titanil sulfate TiOSO_4_, tetraethyl orthotitanate, titanium tetraisopropoxide Ti(OH)_2_ [62,63,64,65,66].

In some cases, the surface of the nanoparticles is modified using polymers, such as bacterial cellulose or chitosan [67,68]. The addition of polymers is intended to reduce the aggregation of NPs in the colloid. In the case of TiO_2_ NPs, studies on surface modification with polymers are relatively rare. Apparently, TiO_2_ NPs are quite stable without surface modification. Separately, it is worth noting the chemical synthesis by precipitation in a medium of plant extracts (*Aloe barbadensis*, *Avicennia Marina*, *Caesalpinia pulcherrima*, *Citrus sinensis*, *Nervilia aragoan* etc.), mushrooms (*Pleurotus djamor*), or bacterial components (*Staphylococcus aureus*, *bacillus subtilis*, etc.) [64,66,69,70,71,72]. This approach is called “green synthesis”. Green synthesis is often called biosynthesis; however, this is erroneous since the method is usually a modification of classical chemical methods, the sol−gel method, the hydrothermal method, the microwave method, etc. [66,73]. However, in the case of synthesis using bacterial culture in a medium with the addition of Ti-containing precursors, the term “biosynthesis” can be applied if the growth of NPs inside the bacteria is proven. An example is the absorption of Ti^3+^ ions by bacteria, their enzymatic reduction, and the growth of NPs inside the bacterial cell [74].

The second approach is usually represented by physical methods of synthesis, which consist of breaking down a large target into micro- and nanoparticles, for example, ball milling technique, physics vapor deposition, and laser ablation of anatase or rulite in a liquid [75,76,77,78,79]. In the case of physical synthesis methods, surface modification with biologically active compounds, such as antibiotics, is also possible [75].

Green synthesis methods are traditionally considered to be more environmentally friendly and cheaper than classical physical and chemical methods. The former requires the use of chemical reagents that can pollute the environment. Physical methods can require high electricity costs and/or expensive equipment [80]. However, we believe that with the right choice of conditions, a number of physical synthesis methods (e.g., laser ablation) can be cost-effective and require no additional reagents (surfactants or counterions) other than the target [81].

**Figure 3:**
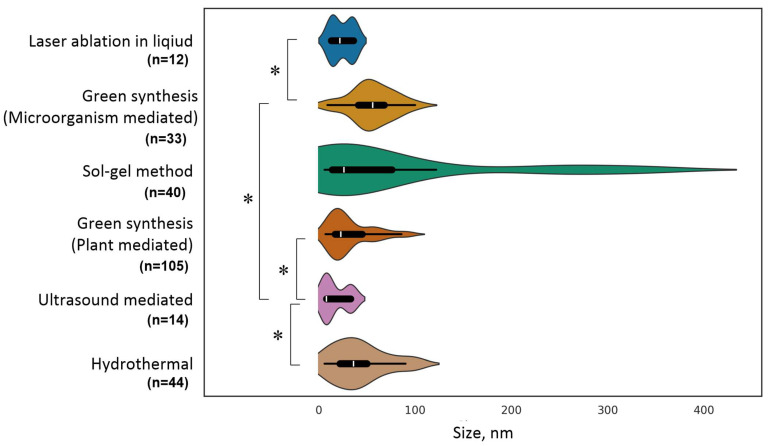
The dependence of TiO_2_ NPs sizes on the synthesis method (references in the text). *—*p* < 0.05, Kruskal−Wallis One Way ANOVA. The sizes of the analyzed samples are shown by numbers in brackets under the corresponding designations of the synthesis methods.

The size of the NPs may depend on the method of their synthesis [82]. We attempted to estimate the dependence of the size of TiO_2_ NPs on the synthesis method (Figure 3). It is worth noting that TiO_2_ NPs have a fairly narrow size distribution. Most NPs have sizes in the range of ~6–200 nm. This spread is much narrower than for a number of other metal NPs and their oxides. For example, the sizes for ZnO NPs ranged from units to thousands of nm [50].

We have found some trends in the change in the sizes of ZnO NPs depending on the synthesis method. First, the NPs obtained by green synthesis methods are larger in size compared to the NPs obtained by chemical (sonochemical) and physical (laser ablation in liquid) methods. The NPs obtained by laser ablation in a liquid have a narrower size distribution than the NPs obtained by chemical methods. TiO_2_ NPs obtained by the hydrothermal method are greater than those obtained by the ultrasonic method.

The antimicrobial activity of NPs may depend on their size; therefore, knowledge of the method-size-antimicrobial activity relationship will allow the future selection of the most optimal methods for synthesizing TiO_2_ NPs.

## 3. Methods for Studying the Physical and Chemical Properties of TiO_2_ NPs

The methods for studying the physical and chemical properties of TiO_2_ NPs are the same as for other NPs. NPs are classically characterized by morphology (size, shape) and chemical composition.

The assessment of the sizes of NPs can be performed in two principal ways. The first is the assessment of the sizes of “dry” NPs using microscopy methods: transmission electron microscopy (TEM), scanning electron microscopy (SEM), and atomic force microscopy (AFM) [83,84,85]. The advantage of these methods is the ability to simultaneously assess both the size and shape of NPs. The disadvantages are the need for additional sample preparation and the impossibility of determining the size of the hydrate shell by assessing the hydrodynamic radius of NPs in a colloid. The latter is critical since NPs in biological experiments are in colloids.

The second approach is the assessment of “wet” NPs in colloids (most often, aqueous solutions) using the dynamic light scattering method (DLS) and analytical separation on a disk centrifuge [86,87]. The undoubted advantage of the DLS method is the ability to assess the distribution of NPs precisely by hydrodynamic diameter, which gives more accurate information about their real size in colloids, taking into account the hydration shell. In addition, analyzing the distribution of NPs by the value of the ζ-potential is simultaneously possible with measurements by the DLS method. ζ-potential carries additional information about the stability of NPs in colloids to aggregation [86,88]. The method of differential centrifugal sedimentation allows not only to determine their distribution by size (mass) but also to purify interested individual NPs fractions from a heterogeneous population [89]. The disadvantages of the method include the impossibility of obtaining additional information and the duration of execution (hours) [87].

The sizes of NPs can also be investigated using the Coulter principle, which is based on the fact that particles moving in an electric field cause measurable disturbances in the same field. The magnitude of these disturbances is proportional to the size of the particles in the field [90]. A method for measuring the sizes of NPs based on this principle is called ion occlusion scanning (SIOS) [91]. Less common are other methods for assessing the size of NPs: particle size mobility scanning (SMPS) and nanoparticle tracking analysis (NTA) [92,93]. The first method allows us to estimate the size of NPs in aerosol, and the second gives additional information about the diffusion rate of NPs in colloids.

The chemical composition of NPs can be evaluated/validated using several methods. Energy dispersive spectroscopy (EDX) is commonly used to determine the chemical composition [94,95], which is very convenient since this method is usually integrated into modern transmission electron microscopes. X-ray photoelectron spectroscopy (XPS) is also often used [96]. In addition, the crystal structure of nanoparticles is often studied using X-ray diffraction (XRD) [97]. Pure TiO_2_ NPs and their conjugates are usually characterized using UV-Vis absorption spectroscopy. Pure TiO_2_ NPs have prominent absorption peaks at 300 and 370 nm. [75]. Fourier transform infrared spectroscopy (FTIR) is also an informative spectral method [98]. For TiO_2_ NPs, characteristic absorption peaks in the regions of ~3400 cm^−1^ and 1640–1160 cm^−1^ have been described [75,98].

In the case of the inclusion of NPs in composite polymer materials, differential scanning calorimetry and the Brunauer−Emmett−Teller (BET) method are used [99]. This method is used to study the surface area of NPs and the rheological properties of nanomaterials. Modulation interference microscopy (MIM) is used to study the spatial distribution of nanoparticles within the polymer matrix [100,101]. The stability of NPs colloids in a solvent is studied by measuring the ζ-potential, as mentioned above [102]. The magnitude of the ζ-potential (by modulus): >60 mV—excellent stability of NPs colloids, 40–60 mV—good stability, 30–40 mV—average stability, <30 mV—moderate stability and the ability of NPs to aggregate over time, <5—low stability, rapid aggregation of NPs [103,104].

## 4. Factors Determining the Magnitude of the TiO_2_ NPs Antimicrobial Effects

### 4.1. Target Microorganism

**Figure 4:**
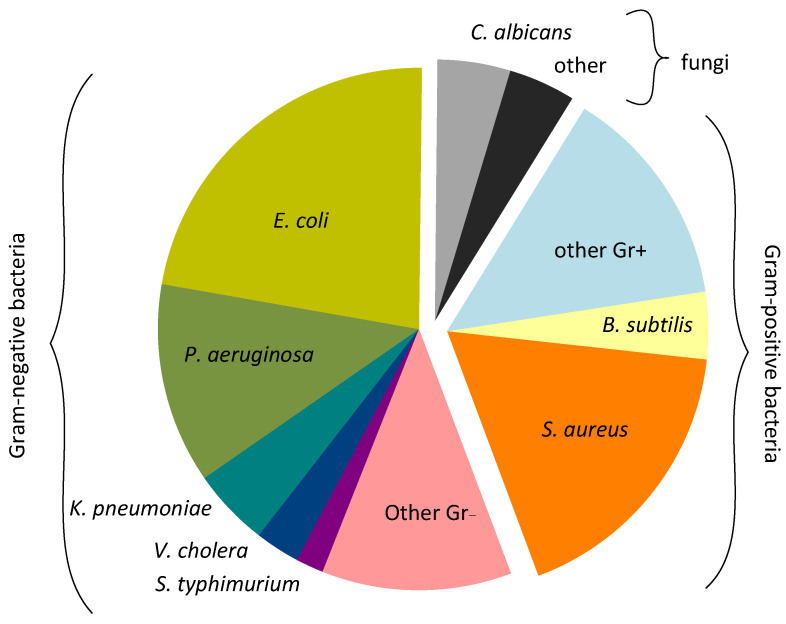
Frequency of occurrence of the studied microorganism species in the analyzed articles (references in the text).

The antimicrobial effects of TiO_2_ NPs against a fairly wide range of microorganisms of epidemiological significance are described, including Gram-negative (Gr−), Gram-positive (Gr+) bacteria and fungi. The most frequently encountered microorganism types in the analyzed works and the proportions of publications in which they were studied are shown below (Figure 4). In the analyzed works, the bacteriostatic effect was most often shown against Gram-negative bacteria (in total ~56%), while Gram-positive bacteria were studied less frequently (in total ~34%). Studies of antifungal potential were much less common (~9%).

Among Gram-negative microorganisms, the antibacterial effect of TiO_2_ NPs against *Escherichia coli*, *Pseudomonas aeruginosa*, and *Klebsiella pneumonia* was often described. *Vibrio cholerae* and *Salmonella enterica serotype typhimurium* were studied less commonly. Among Gram-positive bacteria, the most frequently studied species were *Staphylococcus aureus* and *Bacillus subtilis.* All of the listed bacterial species are of high epidemiological significance, and many of them are characterized by the presence of antibiotic-resistant strains [71,105,106,107,108,109,110,111]. Among fungi, special attention is paid to the causative agent of thrush *Candida albicans* (about half of all analyzed works on antifungal action). Infections caused by antifungal drugs-resistant *C. albicans* strains are especially dangerous in immunodeficiency states [112]. In this case, the search for alternative methods of combating fungal infections becomes a priority.

There are emerging data on the antiviral activity of TiO_2_ NPs, in particular against the H_3_N_2_ influenza virus [113,114].

The greater number of publications indicating antibacterial action against Gram-negative bacteria may indicate potentially greater effectiveness of TiO_2_ NPs against this particular group of bacteria. Higher effectiveness against Gram-negative bacteria may be of interest since, due to the peculiarities of the structure of the cell wall, it is among them that aggressive antibiotic-resistant forms are more common [115].

**Figure 5:**
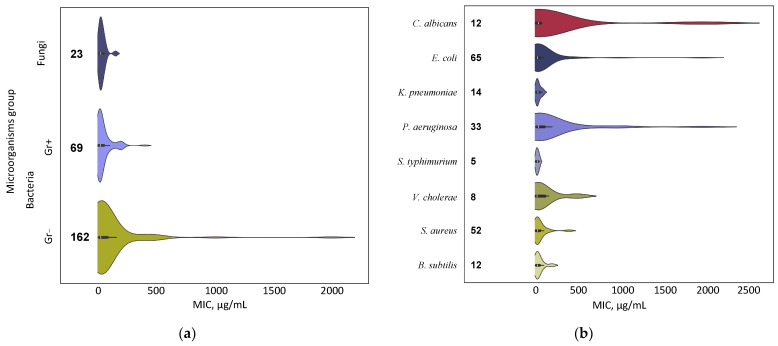
Dependence of MIC of TiO_2_ NPs from group (**a**) and species (**b**) of microorganisms. The data are presented as distribution histograms with box plots. Sample sizes are indicated to the left of the corresponding distribution histograms. Different colors represent different groups (**a**) or species (**b**) of microorganisms.

We decided to compare the dependence of MIC on the studied microorganism group to test our hypothesis (Figure 4). First, we divided all microorganisms into large groups: Gram-positive, Gram-negative bacteria, and fungi (Figure 5a). One can see a tendency for MIC to depend on the group: Gram-positive bacteria appear to be more susceptible to TiO_2_ NPs than Gram-negative bacteria and fungi. However, we did not find any statistically significant differences when testing statistical significance using the Kruskal−Wallis One Way ANOVA and Mann−Whitney Rank Sum test.

When assessing the dependence of MIC on the type of microorganism, no statistically significant differences were found among epidemiologically significant ones (Figure 5b). However, for a number of microorganisms, *Candida albicans*, *E. coli,* and *P. aeruginosa* in some of the works, a shift of MIC distribution towards higher values can be detected, which may indicate significant differences in sensitivity to TiO_2_ NPs between different strains of the same species. For *S. aureus* and *B. subtillis*, the opposite picture was observed: a narrow shape of the distribution, indicating small differences in sensitivity to TiO_2_ NPs between strains.

The weak dependence of MIC on the species of microorganisms may indicate a number of features of NPs. Firstly, the universality of their antimicrobial action and a wide range of potential applications. Secondly, there are other factors (shape and size, presence and type of dopants, etc.) that can determine the magnitude of antimicrobial activity to a greater extent than the type of microorganism. Thirdly, the weak dependence of MIC of TiO_2_ NPs on the type of microorganism under study allows us, if necessary, to conduct a further search for factors determining the antimicrobial potential without taking into account the systematic affiliation of microorganisms.

### 4.2. Morphology of NPs

**Figure 6:**
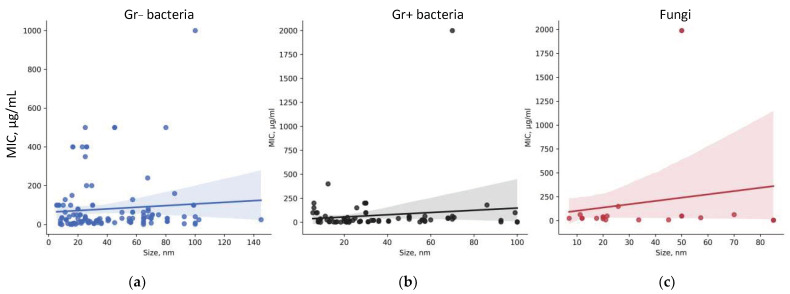
Dependences of MIC on the TiO_2_ NPs size for Gram-negative (**a**), Gram-positive (**b**) bacteria, and fungi (**c**). The dots indicate individual values in the size-MIC pairs taken from the published works. The straight lines correspond to the trend lines; the shaded areas correspond to the confidence interval of 0.95.

The influence of NPs morphology (shape and size) on their antibacterial activity has been shown for NPs of many other metals and their oxides, for example, ZnO, Ag_2_O, iron oxides, and others [50,51,116,117,118]. The most obvious dependence of MIC on the size of NPs and their shape seems to be. A decrease in the size of NPs should increase the ratio “surface area/volume (S/V)” and increase their biological activity [119]. We did not find any noticeable trends in the average assessment of the size-MIC dependence, so we then assessed the dependence of MIC on the size of NPs for different groups of microorganisms (Figure 6).

**Figure 7:**
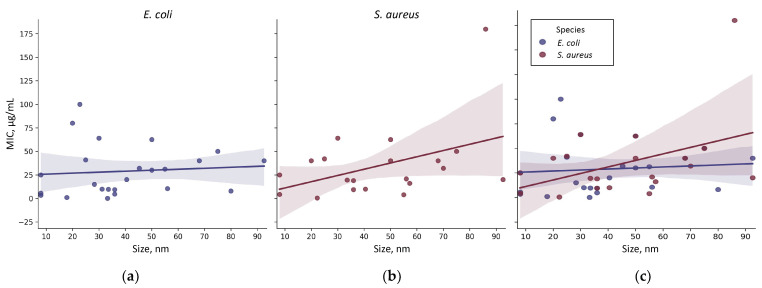
Dependences of MIC on the size of TiO_2_ NPs for *E. coli* (**a**) *S. aureus* (**b**) shown separately and on one graph (**c**). The dots indicate individual values in the size-MIC pairs, taken from the published works. The straight lines correspond to the trend lines; the shaded areas correspond to the confidence interval of 0.95.

For all groups, a tendency towards a decrease in MIC and, consequently, an increase in antimicrobial activity with decreasing size is observed. This tendency corresponds to the classical concept described in the literature for other NPs [120,121,122]. In the case of fungi, this tendency is more pronounced (Figure 6c). It is also worth noting that at sizes <20 nm for Gram-negative bacteria and <5 nm for Gram-positive bacteria, an increase in MIC is observed. Thus, the size-MIC dependence has a complex shape, and the optimal size of NPs with the highest antibacterial activity is 40–60 nm and 10–30 nm for Gram-negative and Gram-positive bacteria, respectively. Perhaps the weak expression of the detected dependencies still depends on the type of microorganism. For further analysis, we constructed the size-MIC dependences for the most frequently encountered types of Gram-negative and Gram-positive bacteria in the works: *E. coli* and *S. aureus*, respectively (Figure 7).

The dependence of MIC on the size of TiO_2_ NPs differs between microorganisms: pronounced for *S. aureus* and practically absent for *E. coli*. However, in the size range of 20–50 nm, the MIC for both bacteria is practically the same. The weak, pronounced dependence of the MIC of TiO_2_ NPs (R^2^~3) on the species of the microorganism under study allows us to conduct further searches for factors determining antimicrobial potential without taking into account the systematic affiliation of microorganisms.

**Figure 8:**
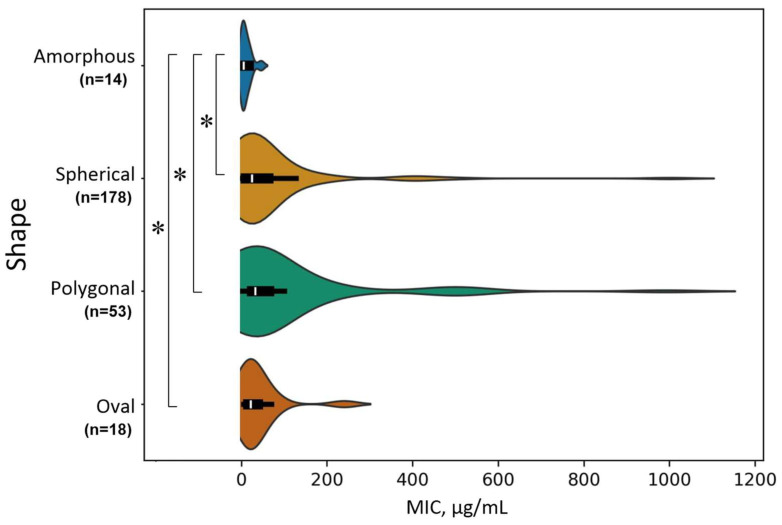
Dependence of MIC on the form of TiO_2_ NPs. *—*p* < 0.05, Kruskal−Wallis One Way ANOVA. The volumes of the analyzed samples are shown by numbers in brackets under the corresponding descriptions of the forms. Different colors represent different groups (a) or species (b) of microorganisms.

The second important parameter determining the antimicrobial activity and cytotoxicity of NPs is their shape [123,124,125]. TiO_2_ NPs can take several different forms depending on the conditions used for their synthesis. We identified the most common groups of shapes: spherical, oval (ellipses and rods), polygonal (including tetragonal, cuboid, hexagonal, and quasi-spherical), and amorphous. When choosing the shape, we relied on such criteria as the presence/absence of angles and the ratio of length to width. The result of comparing the MIC in the groups by shape is shown below (Figure 8). Polygonal, spherical, and oval NPs have high MIC values compared to amorphous NPs. Amorphous TiO_2_ NPs demonstrate a more pronounced antimicrobial effect compared to other forms. For amorphous NPs, the expected surface area/volume (S/V) ratio with equal dimensions will be greater than for formed ones (especially compact ones: spheres or quasi-spheres) [126]. Consequently, the value of the S/V ratio significantly affects the antimicrobial activity of TiO_2_ NPs.

### 4.3. Modification of the Composition of NPs

**Figure 9:**
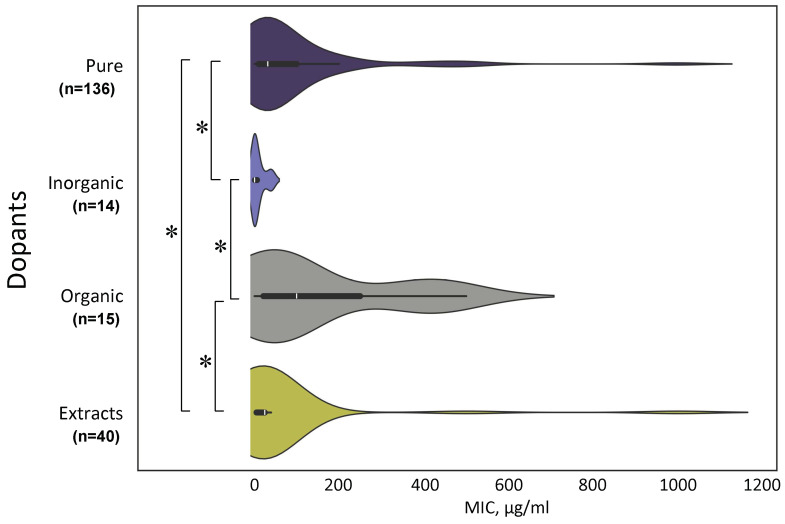
Dependence of MIC on the method of LF modification. *—*p* < 0.05, Kruskal−Wallis One Way ANOVA and Mann−Whitney Rank Sum Test. The volumes of the analyzed samples are shown by numbers in brackets under the corresponding descriptions of the forms.

Modifications of the surface and/or composition of NPs using inorganic and/or organic compounds (antibiotics, polymers, components of plant extracts, and bacterial cultures) are the main ways to increase the antimicrobial activity of NPs [127,128,129,130,131,132]. It is difficult to cover the entire variety of methods for modifying NPs within the framework of one review. However, we attempted to combine all the modifications we encountered in the literature into four general groups based on the type of dopant introduced into the NPs: (1) unmodified (Pure); (2) inorganic dopants (metals and non-metals); (3) organic dopants of known composition (antibiotics, polymers); (4) components of plant extracts modifying NPs as a result of “green synthesis” (Figure 9).

We have found that the greatest antimicrobial effect is exhibited by TiO_2_ NPs modified with inorganic compounds: metals (Ba, Ag, Nd, and Mg) or non-metals (sulfur) [60,133,134,135,136]. Pure TiO_2_ NPs and those modified with organic compounds have less pronounced antimicrobial activity. TiO_2_ NPs modified with components of plant extracts or components of bacterial nature have an intermediate activity between pure and modified inorganic compounds of TiO_2_ NPs.

## 5. Mechanisms of Antimicrobial Action, Photocatalysis and Characteristics of Excitation Light

The mechanisms of antimicrobial action for the vast majority of metal oxide PMs are the release of metal ions, Fenton reaction in the case of variable valences metals and direct destruction of the cell wall by adhesion of NPs to cells, mutagenic and genotoxic action, enzyme inactivation and photocatalysis [50,51,117]. Unlike most metal oxide NPs, for TiO_2_ NPs, the most pronounced and studied mechanism of antibacterial action is photocatalysis [137].

The second mechanism of antimicrobial action is the enhancement of the effectiveness of antimicrobial compounds, in particular antibiotics, as exemplified by amoxicillin [75]. This effect is logical since TiO_2_ NPs have excellent structure for creating targeted drug delivery systems [113,138,139].

The third mechanisms of toxicity of TiO_2_ NPs are genotoxicity and mutagenicity, but the exact mechanisms are still unknown [140].

**Figure 10:**
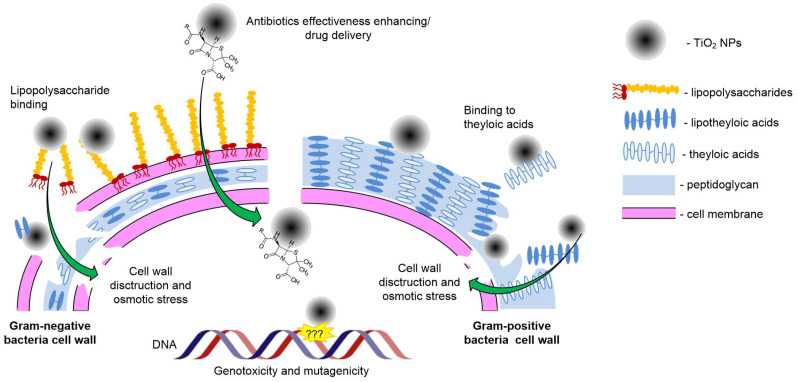
Generalized scheme of non-photocatalytic antibacterial actions of TiO_2_ NPs (references in the text).

Antimicrobial activity in the dark through disruption of the cell wall integrity has also been shown in principle; however, in the presence of light, the process of cell wall lysis is accelerated. The addition of carbon quantum dots significantly enhances the photocatalytic toxicity of TiO_2_ NPs against bacteria [141]. It has been shown that TiO_2_ NPs when in contact with the outer cell membrane of Gram-negative bacteria, are capable of causing its depolarization, which entails disruption of the membrane barrier function [142]. The molecular targets of TiO_2_ NPs, in this case, are membrane proteins, lipopolysaccharides (Gram-negative bacteria), and lipoteichoic acids (Gram-positive bacteria) [40,143]. Osmotic stress has also been described as a mechanism of dark toxicity against bacteria [144]. The generalization of ‘dark’ non-photocatalytic mechanisms of antibacterial activity of TiO_2_ NPs is presented in Figure 10.

The reason for the high photocatalytic activity of TiO_2_ NPs is the ability of TiO_2_ to absorb light and fluoresce in wide wavelength ranges. Pure TiO_2_ absorbs light in the range of λ 200–300 nm and releases light with wavelengths from 400 to 700 nm. [145,146]. The broadening of the absorption region of TiO_2_ NPs up to 520 nm can be observed when organic compounds capable of fluorescence (e.g., indole) are added to TiO_2_ NPs [145]. Enhancement of absorption in the IR region (according to FTIR data) for TiO_2_ NPs in the presence of indole has also been shown.

Light can interact with TiO_2_ NPs and lead to the formation of ROS such as superoxide anion radical O_2_^−•^ and hydroxyl radical ^•^OH [147]. TiO_2_ has a very high wide value band gap of 3.2 eV, due to which, upon absorption of UV radiation (optimally, ≤385 nm), electron-“hole” pairs with high energy are formed. The energy of electron-“hole” pairs can then be transferred to surrounding molecules [148].

The generalized equations of the processes of ROS generation on TiO_2_ NPs surface during photolysis are shown below:

TiO_2_ + *hv*_(UV−visible)_ → TiO_2_ + (*e*_CB−_) + *h*_VB+_ (1)

*e*_CB−_ + O_2_ → O_2_^−•^ (2)

**Figure 11:**
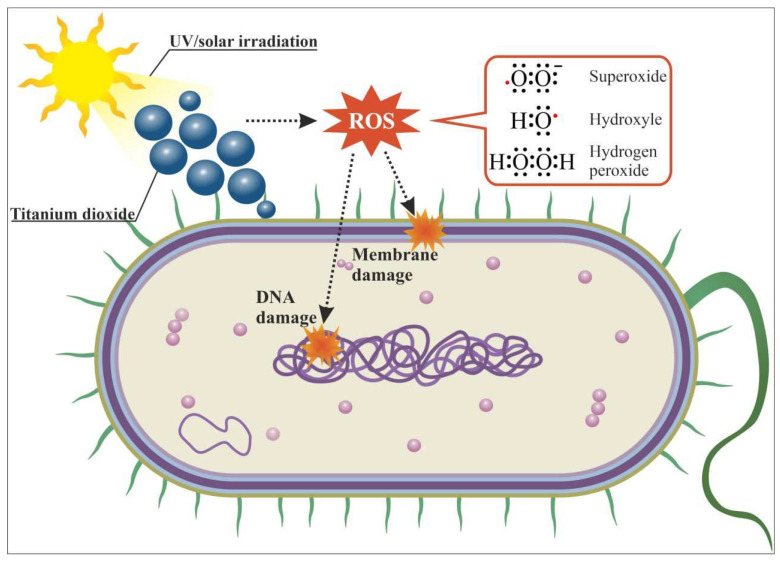
Generalized scheme of photocatalytic antibacterial action of TiO_2_ NPs [149,150,151,152,153].

*h*_VB+_ + OH^−^ → ^•^OH (3)

where CB is the conduction band, VB is the valence band, *h*_VB+_ is the electron “hole”, *e*_CB−_ is the “knocked out” photon electron. If photocatalytic transformations formed ROS (Figure 11) significantly accumulated in the cell in concentrations exceeding the limit of the antioxidant system, the ROS causes oxidative stress [149,150]. During oxidative stress, chemical modifications of both protein molecules and DNA occur [151,152,153].

The first can lead to disruption of enzyme activity. The second leads to genotoxicity, which disrupts DNA duplication processes. ROS are also capable of damaging the structure of bacterial cell walls and destroying bacterial biofilms [86].

Unfortunately, not all articles indicate the wavelengths of light in the study. In addition, the authors do not indicate the radiation power, which makes it difficult to compare the results with each other. However, we will try to describe the main points.

The first is the wavelength. A decrease in light wavelength significantly enhances the antimicrobial (bacteriostatic) effect of TiO_2_ NPs: a change in λ from 425 to 365 nm reduced the number of CFU *E. coli* by 3.5 orders of magnitude [154]. TiO_2_ NPs, in combination with UV-B (~280 nm), cause significant inhibition of the expression of bacterial genes responsible for DNA replication, cell division, toxin detoxification, metabolism, ion transport, and others [137]. However, UV-B without TiO_2_ NPs has a significantly smaller effect on the expression of bacterial genes.

The second is the presence of organic compounds. TiO_2_-induced photocatalysis can successfully occur at wavelengths of ~555 and ~608 nm after the addition of organic compounds, such as dyes [155]. It is noteworthy that TiO_2_ NPs modified with compounds of *Achyranthes aspera* extract, on the contrary, can act as antioxidants in the dark [155]. Therefore, both the presence of light and the properties of the chemical environment are important for the implementation of the photocatalytic activity of TiO_2_ NPs.

Third is a production method. The method of production of TiO_2_ NPs and “freshness” of NPs also influence their photocatalytic activity. For example, TiO_2_ NPs obtained by laser ablation just before the test had a more pronounced UV-induced bacteriostatic effect than commercially available NPs obtained by chemical methods [156].

**Figure 12:**
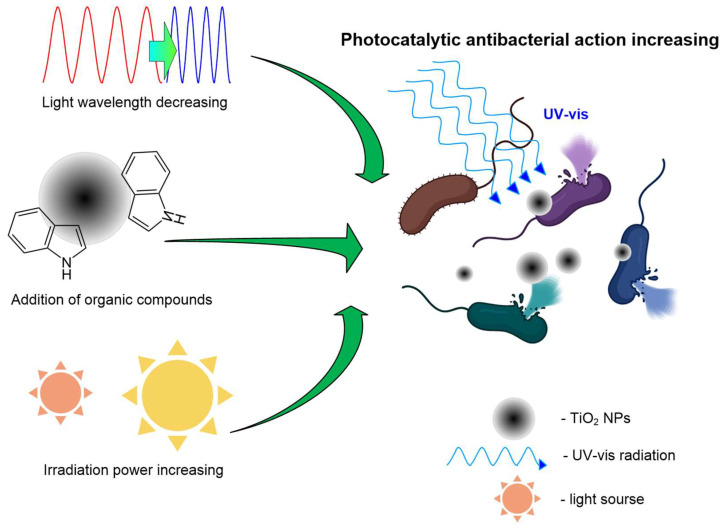
Main methods of photocatalytic antibacterial action of TiO_2_ NPs improving (reference in the text).

Fourth is the radiation power (Figure 12). Higher irradiation power naturally increases the antibacterial activity of TiO_2_ NPs, e.g., the MIC for UV powers of 11 W/m^2^ and 30 W/m^2^ are 25 and 1 µg/mL, respectively [156,157]. The shape of the NPs can also affect the efficiency of photocatalysis. For example, the MIC for amorphous TiO_2_ NPs is lower than for spherical ones [17,157].

Thus, the photocatalytic activity of TiO_2_ NPs may depend on the wavelength of the exciting light, its power, NP morphology, and the presence of organic compounds capable of fluorescence or light absorption in the NP colloid. However, it should be noted that the diversity of experimental conditions and their modifications in published works complicates the unambiguous interpretation of the analyzed data.

Other methods for increasing the antimicrobial activity of TiO_2_ NPs usually involve modification of classical synthesis methods. Laser ablation in a constant magnetic field of 1.2 T allowed the synthesis of TiO_2_ NPs with 20–30% greater bacteriostatic activity than without the use of a magnetic field [158]. Modification of the surface of nanoparticles with polymers in the case of TiO_2_ increases their bacteriostatic effect by 50–100% compared to untreated nanoparticles. This effect is realized in the dark [67]. TiO_2_ NPs synthesized in a modified atmosphere with 50% or 75% argon showed more pronounced bacteriostatic properties than TiO_2_ NPs synthesized in a normal atmosphere [159]. The combined use of antibiotics and TiO_2_ NPs can provide a synergistic bacteriostatic effect even against antibiotic-resistant strains [160]. TiO_2_ NPs modified with silver and components of pyrolyzed diatomaceous algae biomass made it possible to achieve a significant bacteriostatic effect against Gram-positive *S. aureus* and Gram-negative *K. pneumoniae* and *E. coli* bacteria both in laboratory tests and in the case of clinical isolates [161].

Modification with plant extract components can enhance the antimicrobial and antibiofilm activity of TiO_2_ NPs via capturing of AL-2 QS signaling molecules and strong oxidative stress, which in turn causes disturbances in enzyme activities, protein and nucleic acid integrity, and biofilm matrix [162].

As mentioned earlier, although TiO_2_ NPs are generally considered titanium dioxide, they are actually a mixture of rutile and anatase in different ratios [75]. Rutile and anatase have slightly different O/Ti stoichiometry, namely 1.9 and 2.0, respectively [37]. Increasing the proportion of rutile compared to anatase in the NPs mixture (reducing the O/Ti stoichiometry from 2.0 to 1.9) was found to enhance the antibacterial activity against both Gram-negative and Gram-positive bacteria due to enhanced photocatalysis [163]. Microcoatings containing individual rutile crystals in anatase also demonstrated good antibacterial potential both in the dark and under visible UV light illumination [164]. The authors of the work attribute the increase in antimicrobial activity to the larger surface area of rutile crystals than anatase. A more pronounced antimicrobial effect against *E. coli* and *S. aureus* of rutile/anatase NPs compared to “pure” anatase NPs is also found in a number of studies [75,95]. It is worth noting that the synthesis of titanium oxide-based NPs and nanocoatings with a given O/Ti stoichiometry is a complex methodological task that requires careful selection of synthesis conditions, precursor ratios during synthesis, and/or surface modification [163,164].

## 6. Biocompatibility with Eukaryotic Cells

Despite the canonical view of TiO_2_ NPs as biosafe for eukaryotic cells, a wealth of data are emerging on the potential cytotoxicity of TiO_2_ NPs [165]. The dependence of TiO_2_ NPs cytotoxicity against hamster lung fibroblasts on NPs geometry was shown [79]. The mechanism of cytotoxic action includes genotoxic action induction of cell apoptosis [79].

It is worth noting that cytotoxic concentrations for eukaryotic cells of ~100 μg/mL can be in the order of magnitude of the average MIC of TiO_2_ NPs for microorganisms [79]. The TiO_2_ NPs’ cytotoxicity increased with decreasing of size in the range from 10 to 170 nm [79]. The dependence of cytotoxicity on the size of NPs is well illustrated by the following example.

As a rule, cytotoxicity rises with increasing NPs concentration (from 0.1 to 100 μg/mL) and exposure time (from 1 to 4 days) [166]. On the other hand, it was shown on A549 lung cells that TiO_2_ NPs prepared by different methods with large sizes of ~100 and ~1000 nm do not have a toxic effect on cells. They did not cause cell death, DNA fragmentation, or oxidative stress or change the activity of dehydrogenases and the concentration of glutathione [167]. The only alarming fact is the ability of TiO_2_ NPs to penetrate into the cell and accumulate there, which was shown by TEM methods [167]. However, no experimental evidence of cell death and/or cell cycle modification from the accumulation of TiO_2_ NPs in the cell has been found.

In addition, the TiO_2_ NPs cytotoxicity depends on the Ti^4+^, Ti^3+,^ and Ti^2+^ ratio in NPs and the type of cells studied. In particular, TiO_2_ NPs did not affect the viability of rat fibroblasts but caused the death of rat erythrocytes through oxidative stress and calcium overload. NPs with a higher Ti^4+^/Ti^3+^ ratio caused more pronounced oxidative stress and Ca^2+^ influx into erythrocytes [168]. Differences in the cytotoxic concentrations of TiO_2_ NPs were shown for different hepatocyte lines: SMMC-7721 human hepatocarcinoma, normal human liver cells HL-7702, rat tumor cells CBRH-791, and normal rat cells BRL-3A. Rat cells were more sensitive to TiO_2_ NPs. TiO_2_ NPs showed greater toxicity and ROS production in the case of hepatocarcinoma cells of both species compared to normal cells [166]. Therefore, TiO_2_ NPs have potential as an anticancer agent, but, in our opinion, additional methods are required to increase their specificity. Work in this direction is underway. In particular, the addition of silver TiO_2_ NPs increases their cytotoxicity against human hepatocarcinoma HepG2 cells due to disruption of mitochondrial function and induction of apoptosis. [169].

To summarize the above, the cytotoxicity of TiO_2_ NPs depends on a wide range of conditions: concentration, time of action, shape and size of the NPs themselves, their composition modifications, and cell type. TiO_2_ NPs can be used as antimicrobial and anticancer agents with the right combination of conditions.

## 7. Prospects and Limitations

In this review, we attempted to evaluate the dependence of the antimicrobial activity of TiO_2_ NPs on their characteristics (morphology, composition, synthesis method), as well as on the systematic position of microorganisms. It was somewhat unexpected for us that the antibacterial properties were very weakly dependent on the Gram’s count of the bacteria and were little dependent on the species. The dependence of MIC on the size of TiO_2_ NPs was observed but was weakly expressed. The optimal size of TiO_2_ NPs is 20–100 nm when MIC is minimal. A more pronounced effect on antimicrobial activity was exerted by such factors as the surface modification of TiO_2_ NPs and, consequently, the synthesis method, as well as the shape of the NPs. The most pronounced antimicrobial activities were shown by amorphous TiO_2_ NPs or modified with other metals, such as Mg, Ag, etc. The key mechanism of the antimicrobial activity of TiO_2_ NPs is photocatalytic activity. According to the analyzed literature data, the efficiency of photocatalysis against bacteria and fungi depends on the wavelength of the exciting light, its power, NPs morphology, and the presence of organic compounds in the NP colloid capable of fluorescence or light absorption. However, it should be noted that the diversity of experimental conditions and their modifications in published works complicates the unambiguous interpretation of the analyzed data.

In most studies, the assessment of antibacterial activity was performed using standardized microbiological methods based on the optical density of the suspension, the number of colony-forming units (CFU), and the size of the inhibition zone. [154,160]. For the cultivation of microorganisms, the authors of the works used classical media MHA, BHI, SDA, and a number of others and conditions close to the human body (37 °C) [170,171,172,173].

However, the difference in the methods of culturing bacteria and fungi and assessing the antimicrobial potential complicates the unambiguous interpretation of the obtained dependences. In some studies, the authors separately determined MIC using serial dilutions (Table 1, work numbers are in bold) [136,174]. In other studies, the authors did not directly indicate MIC, but it can be determined based on graphical and/or digital/tabular data in the articles. In the second case, the accuracy of MIC determination may be low and limited to the lower value of the concentration of the studied NPs. This limitation of the accuracy of MIC determination in a number of studies requires special care when searching for quantitative patterns. For example, this may mean that in studies without indicating a separate determination, MIC itself may be artificially inflated. In future studies, it may be worth adding additional criteria for analyzing MIC dependencies, such as the influence of the cultivation environment. However, it is worth remembering that the introduction of additional conditions may not clarify but, on the contrary, “confuse” the researcher. Therefore, the addition of new analysis conditions must be weighed and justified.

In this paper, we attempted a fairly simple and obvious quantitative analysis: assessing the possible relationship between a pair of features. In the future, for a more correct analysis, it may be possible to use multivariate analysis methods, such as the principal component method, multiple correlation, and others.

The final limitation of TiO_2_ NPs application is the development of bacterial resistance against metal NPs and their oxides. Among the main mechanisms of NP resistance described in the literature are activation of ion pumps (protection against free ions), electrostatic repulsion, changes in cell morphology, biofilm formation and modification of the extracellular matrix (protection against contact toxicity), gene transfer, metabolic reactions and mutations (protection against genotoxic action and oxidative stress) [40,175]. In the case of TiO_2_ NPs, it is necessary to overcome the bacterial defense mechanisms against contact and ROS-mediated action. The search for ways to overcome bacterial resistance against NPs should become one of the most pressing tasks in this area.

**Table 1:** Parameters of TiO_2_ NPs reported in the literature.

**No.**	Synthesis Method	Composition	Size, nm	Shape	MIC	Microorganism		Medium, Conditions	Effect	Notes	Ref.
						**Group**	**Species**				
1	Laser ablation in water	TiO_2_ amoxicillin + TiO_2_	11–26 (200 mJ; 10 min) 2–23 (80 mJ, 20 min)	Sph	400 µg/mL >> >> >>	Gr− Gr− Gr− Gr+	*E. coli*, *P. aeruginosa* *P. vulgaris*, *S. aureus*	NA, 24 h, 37 °C	BS	Amoxicillin addition significantly increased antibacterial activity.	[75]
**2**	*Aeromonas hydrophila* mediated biosynthesis	TiO_2_	28–54 40.50 (individ.)	Sph	25 µg/mL 20 µg/mL 30 µg/mL 10 µg/mL 10 µg/mL 15 µg/mL	Gr− Gr− Gr− Gr+ Gr+ Gr+	*A. hydrophila*, *E. coli*, *P. aeruginosa*, *S. pyogenes*, *S. aureus*, *E. faecalis*	NA, 24 h, 37 °C	BS	The antibacterial activity of the synthesized TiO_2_ NPs was assessed by well diffusion method toward *A. hydrophila*, *E. coli*, *P. aeruginosa*, *S. aureus*, *S. pyogenes*, and *E. faecalis* and showed effective inhibitory activity against *S. aureus* (33 mm) and *S. pyogenes* (31 mm).	[95]
**3**	*Aspergillus* flavus mediated biosynthesis	TiO_2_	62–74	Sph, Oval	40 µg/mL 40 µg/mL 80 µg/mL 70 µg/mL 45 µg/mL	Gr+ Gr− Gr− Gr− Gr+	*S. aureus*, *E. coli*, *P. aeruginosa*, *K. pneumoniae*, *B. subtilis*	MHA, 24 h, 37 °C	BS	Fungus-mediated synthesized TiO_2_ NPs have proved to be a good novel antibacterial material.	[98]
4	Sol–gel method	TiO_2_	6(S1)–25.8(S2) 25.8–33(S3)	Sph	150 µg/mL (S1, S2) 200 µg/mL (S3) 200 µg/mL (S1, S3) 200 µg/mL (S1) 150 µg/mL (S1) 200 µg/mL (S3) 150 µg/mL (S2)	Gr+ Gr+ Gr+ Gr− Gr− Gr− Fungus	*S. aureus*, *S. pneumonia*, *B. subtilis*, *P. vulgaris*, *P. aeruginosa*, *E. coli*, *C. albicans*	MHA, 12–24 h, 37 ± 1 °C (bacteria) and 27 ± 1 °C (fungus)	BS	The synthesized TiO_2_ NPs were found to be effective in visible light against *S. pneumonia*, *S. aureus*, *P. vulgaris*, *P*. *aeruginosa,* and *C. albicans*. The powder samples at different calcination temperatures are defined as S1 (400 °C), S2 (600 °C), and S3 (800 °C).	[17]
5	Commercial TiO_2_ from Evonik Industries	LDPE-TiO_2_	25	Sph	500 µg/mL	Gr−	*E. coli*	LB, 24 h, 37 °C	BS, FS	The antimicrobial activity of the TiO_2_ NP-coated films exposed under both types of lighting was found to increase with an increase in the TiO_2_ NP concentration and the light exposure time. The antimicrobial activity of the films exposed under UV light was higher than that under fluorescent light.	[154]
6	Microwave-assisted one-step biosynthesis	TiO_2_	20–40	Sph	30 µg/mL >> >> >> >>	Gr+ Gr+ Gr− Gr− Gr+	*Bacillus*, *S. mutans*, *E. coli*, *K. pneumonia*, *C. absonum*	NA, 24 h, 37 °C	BS	The developed TiO_2_ NPs demonstrate significant antibacterial activity against bacillus at the concentration of 70 mg/mL.	[155]
7	Pulsed laser ablation in water	TiO_2_	34	Sph	25 µg/mL	Gr−	*E. coli*	LB, 12 h. (overnight), 37 °C	BS, BC	The best activity was obtained at the highest TiO_2_ concentration (100 µg/mL) and laser-ablated NPs compared with commercial.	[156]
8	Magnetic Field-Assisted Laser Ablation in Water	TiO_2_	25–35	Sph	-	Gr− Gr+	*E. coli*, *S. aureus*	MHA, 24 h, 37 °C	BS	The antibacterial effect assay revealed the largest inhibition zone in *S. aureus* and *E. coli*, with a more potent effect for TiO_2_ NPs prepared by a magnetic field when compared with that prepared without the presence of a magnetic field.	[158]
9	Commercial TiO_2_ from Jinan Haidebei Bioengineering Co., Ltd. Jinan City, Shandong, China	Chitosan-TiO_2_	30	Sph	100 µg/mL	Gr− Gr+	*E. coli*, *S. aureus*	NA, 24 h, 37 °C	BS	The chitosan-TiO_2_ nanocomposite exhibited an inhibitory effect on the growth of *E. coli* and *S. aureus*. When the TiO_2_ NPs concentration was 0.05%, the maximum inhibition zone was found, 11.37 ± 0.76 mm, indicating that, under this concentration, the treatment showed a strong bacteriostasis effect on *E. coli*.	[67]
10	Hydrolysis of Titanium Tetrachloride (TiCl_4_) as precursor	TiO_2_	70–100	Sph	5.14 µg/mL 5.35 µg/mL	Fungus Fungus	*C. albicans ATCC 10231 (Fluconazole-susceptible)* *C. albicans ATCC 76615 (Fluconazole-resistant)*	YNB, 48 h, 37 °C	FS	Yeast cells of *C. albicans,* due to their thick cell wall consisting of glucan and chitin, are more resistant than bacteria. It was reported that TiO_2_ NPs, by producing intracellular reactive oxygen species, induce destructive effects inside the microbial cells, oxidation of intra-cellular Coenzyme A, and peroxidation of the plenty of lipids, which decrease respiratory activity and subsequently cause cell death.	[54]
11	Sol–gel method	TiO_2_	~100	Amorph	1 µg/mL	Gr−	*E. coli*	MHB, 24 h, 37 °C	BS	The results indicate that in the first 30 min of exposure of the bacteria to the activated amorphous TiO_2_, the presence of *E. coli* colonies was significantly reduced, with no presence being detected in the culture.	[157]
12	Electrochemical method (current density was varied from 10 mA/cm^2^ to 14 mA/cm^2^)	TiO_2_	25–30	Amorph	50 μL ^1^ >>	Gr− Gr+	*E. coli*, *S. aureus*	NB, 24 h, 37 °C	BS	TiO_2_ NPs synthesized at 14 mA/cm^2^ (15.99 nm) exhibited maximum (19.1 mm) bacterial growth inhibition against *S. aureus* and (17.0 mm) against *E. coli* in the form of zone-of-inhibition studies.	[56]
13	Green synthesis using ginger and garlic crude aqueous extracts (in varying proportions)	TiO_2_	23.38–58.64	Sph	10000 µg/mL	Gr+	*S. aureus*	MHA, 12 h. (overnight), 37 °C	BS	Garlic-reduced TiO_2_ NPs at elevated concentration exhibited significantly (*p* < 0.05) improved antibacterial activity against MDR *S. aureus*.	[170]
**14**	Sonochemical method	TiO_2_	8	Sph	6.67 µg/mL 4.32 µg/mL 3.96 µg/mL 2.24 µg/mL 3.46 µg/mL 5.54 µg/mL 21.21 µg/mL 3.79 µg/mL 5.7 µg/mL	Gr− Gr+ Gr+ Gr− Gr− Gr− Gr− Gr− Gr−	*P. aeruginosa*, *S. aureus*, *B. aureus*, *E. aerogenes*, *E. coli*, *MRSA*, *S. marcescens*, *A. baumannii*, *S. flexneri*	MHA, 24 h, 37 °C	BC, AV	Results indicated that Gram-positive bacteria were more susceptible compared to Gram-negative bacteria.	[57]
**15**	Synthesis using propolis extract	TiO_2_	57.3 ± 4	Cub, Rect	16 µg/mL 8 µg/mL 32 µg/mL 32 µg/mL 64 µg/mL 64 µg/mL 128 µg/mL 64 µg/mL	Gr+ Gr+ Fungus Gr− Gr− Gr+ Gr− Gr−	*MRSA*, *S. epidermidis*, *C. albicans*, *K. pneumoniae*, *P. aeruginosa*, *L. monocytogenes*, *P. vulgaris*, *A. baumannii*	-(commercial)	BS	The synthesized NPs had higher antimicrobial activity against Gram-positive bacteria than yeast and Gram-negative bacteria, respectively. *P. vulgaris* was the most resistant strain among the tested microorganisms, while *S. epidermidis* was the most susceptible microorganism.	[176]
**16**	Laser ablation in distilled water (DW) and alcohol	TiO_2_	36	Circular	9.45 µg/mL (DW) 4.72 µg/mL (Alc) 18.91 µg/mL(DW) 9.45 µg/mL (Alc)	Gr− Gr+	*E. coli*, *S. aureus*	BHI, 18–24 h, 37 °C	BS	The mechanism of inhibitory activity of TiO_2_ NPs initiated by laser removal on microorganisms could be by their bond to the cell layer and further infiltration inside or by cooperation with phosphorus-containing mixes like DNA exasperating the replication procedure or, ideally, by their assault on the respiratory chain.	[171]
17	Hydrothermal method	TiO_2_	10	Quasi-Sph	50 µg/mL >> >> >> >> >>	Gr− Gr+ Gr+ Gr− Fungus Fungus	*E. coli*, *S. aureus*, *B. subtilis*, *P. aeruginosa*, *C. albicans*, *A.niger*	MHB, 24 h, 37 °C	BS, Antimicrobial	The green synthesized TiO_2_ NPs had high antibacterial activity against Gram-positive than Gram-negative bacteria.	[86]
18	Hydrothermal method	TiO_2_	5–15	Semisph	100 µg/mL >> >>	Gr+ Gr− Gr−	*MRSA*, *E. coli*, *P. aeruginosa*	LB, 12 h. (overnight), 37 °C	BS	The smallest nanocrystallite size showed a pronounced inhibitory effect and high reduction in the growth rate of bacteria with increasing the concentrations of TiO_2_ nanocrystallites for the three strains of bacteria.	[177]
19	Laser ablation in water	TiO_2_	3–30	Sph	400 µg/mL >>	Gr− Gr+	*E. coli*, *S. aureus*	NB, 24 h, 37 °C	BC	The bacterial cell number was dropped in both types of pathogens, and the inhibition was concentration-dependent.	[178]
20	Sol−gel method	TiO_2_	64.77 ± 0.14	Sph	<1024 µg/mL	Gr−	*25 isolates P. aeruginosa*	MHA, 18 h, 34 °C	BS	It was found that TiO_2_ NPs showed a significant reduction in biofilm formation (96%) and represented superior antibacterial activity against *P. aeruginosa* strains in comparison to titanium dioxide powder.	[179]
**21**	Sol−gel method	TiO_2_	35 (anatase), 65 (rutile)	Sph	30 µg/mL 40 µg/mL	Gr− Gr+	*E. coli*, *S. aureus*	MHA, 24 h, 37 °C	BS	Antimicrobial activity results showed a strong bactericidal effect against Gram-positive bacteria and demonstrated greater sensitivity to TiO_2_ NPs at lower concentrations when compared to Gram-negative bacteria.	[180]
22	Photon-induced method (solar-light photocatalyst anatase TiO_2_ NPs)	TiO_2_	40–50 (anatase) 50–70 (mixed phase) 95–110 (rutile) 130–160 (rutile)	Sph	25 µg/mL >>	Gr+ Gr−	*S. aureus*, *E. coli*	MHA, 24 h, 37 °C	BC	Anatase TiO_2_ NPs (25 nm, 100 μg/mL) demonstrated AB activity against extracellular *S. aureus* with 80% and *E. coli* with 82% killing efficacy.	[59]
23	Green synthesis using mulberry plant extract	Mulberry Plant Extract + TiO_2_	24 (anatase)	Sph	20 µg/mL >>	Gr+ Gr−	*S. aureus*, *E. coli*	MHA, 12 h, 37 °C	BS	The Gr+ bacterial strain is more sensitive due to its weak cell wall membrane.	[181]
**24**	Calcination temperature (500 °C, 900 °C)	TiO_2_ + Geraniol (GER)	300 ± 100 (anatase) 100(rutile)	Amorph	1.125 µg/mL 2.05 µg/mL 1.55 µg/mL	Gr+ Gr+ Gr−	*S. aureus CCM 4223*, *MRSA CCM 7110*, *E. coli CCM 3954*	MHB, 24 h, 37 °C	BC	GER enhances antimicrobial properties due to its high solubility and controlled toxicity.	[182]
25	Hydrolysis precipitation method with Ti(OBu)_4_, silver nitrate and ammonia	TiO_2_ N-TiO_2_ Ag-N-TiO_2_	19.8 39.2 20.7	Sph	5000 µg/mL >>	Gr− Gr+	*E. coli* *B. Subtilis*	MHA, 24 h, 37 °C	BS	Both Ag- and N-doped TiO_2_ show increased antibacterial properties of TiO_2_ NPs under fluorescent light irradiation	[62]
26	Biosynthesis by Using *Streptomyces* sp. HC1	TiO_2_	30–70	Sph	<2000 µg/mL >> >> >> >>	Gr+ Gr− Fungus Fungus Gr−	*S. aureus ATCC 29213*, *E. coli ATCC 35218*, *C. albicans ATCC 10231*, *A. niger ATCC 6275*, *P. aeruginosa ATCC 27853*	MHA (bacteria) SDA (fungus) 37 °C for 24 h	BS	TiO_2_ NPs showed higher antimicrobial activity against bacteria (12 mm) than against fungi.	[173]
27	Sol−gel method	TiO_2_ Mg-doped: 0.014 g/mL 0.028 g/mL 0.042 g/mL	9.7 7 6.8 6.5	-	14,000 µg/mL 14,000 µg/mL 42,000 µg/mL	Gr− Gr+ Gr−	*E. coli* *B. subtilis* *Pseudomonas*	-	BS, Antimicrobial	The 0.14 gm Mg solution doped TiO_2_ NPs were found to be a good antibiotic compared to others against the *E. coli* and *B. subtilis* bacteria.	[135]
**28**	Sol–gel electrospinning technique	TiO_2_	200–300	Rods	5 µg/mL >> >> >>	Gr+ Gr− Gr− Gr−	*S. aureus* *E. coli* *S. typhimurium* *K. pneumonia*	NA or TSB, 24 h, 37 °C	BC	The mechanisms by which the UV light-induced photocatalytic activated TiO_2_ nanorods kill bacteria are suggested to be initial oxidative damage to the cell wall and the cell membrane, followed by damage to the interior DNA molecules, eventually causing death.	[172]
29	Ionic liquid-assisted hydrothermal method	TiO_2_	35 (TEM)	Sph	10,000 µg/mL >> >> >>	Gr− Gr− Gr− Gr+	*K. aerogenes* *E. coli* *P. desmolyticum* *S. aureus*	NA, 24–36 h, 37 °C	BS	TiO_2_ nanoparticles exhibited excellent photocatalytic activity for the degradation of methylene blue organic dye.	[183]
30	Two-step sol−gel method using citric acid and alpha dextrose as double surfactants	TiO_2_	20		>500 µg/disc >>	Gr+ Gr+	*S. aureus* *MRSA*	NB, 24 h, 30 °C	BS	In the case of nalidixic acid, TiO_2_ nanoparticle showed a Synergic effect on the antibacterial activity of this antibiotic against the test strain	[160]
31	Sol−gel method	TiO_2_ Nd-TiO_2_	14–10	Sph	40 µg/mL >>	Gr− Gr+	*E. coli* *S. aureas*	NA, 24 h, 37 °C	BS	Neodymium (Nd) doped TiO_2_ enhanced the photocatalytic activity. Nd-doped TiO_2_ NPs showed good antibacterial activity when compared with TiO_2_ nanoparticles.	[134]
32	Hydrothermal method	TiO_2_	<100 (FE-SEM)	Sph	100 µg/mL >> >> >>	Gr− Gr− Gr− Gr+	*P. eruginosa (ATCC^®^ 10145)*, *E. coli (ATCC^®^ 33876)*, *K. pneumonia (ATCC^®^ BAA-1144)*, *S. ureus (ATCC^®^ 11632)*	NA, 12 h. (overnight), 37 °C	BS	TiO_2_ NPs are found to have the maximum antibacterial activity against Gram-negative bacterial strains rather than Gram-positive bacteria. TiO_2_ NPs have been shown to prevent or destroy bacterial cells by adhering to the cell wall, causing the leakage and damage of intracellular contents, hydroxyl radicals, the generation of reactive oxygen species, and the release of Ti_4_^+^ ions.	[184]
33	Hydrothermal post-treatment of amorphous titania at different temperatures (250 °C or 310 °C) without using any additives or doping agent	TiO_2_	~10–80	Sph, faceted	500 µg/mL >> >> >>	Gr− Gr− Gr− Gr−	*A. vitis* *E. amylovora* *P. syringae* *X. juglandis*	LB agar plates, 48 h, 30 °C	BS	Under UV-irradiation, A310C (310 °C) showed a pronounced antimicrobial activity on all the investigated plant pathogenic bacteria. The kinetic curves reveal that the order of susceptibility of tested bacteria using A310C is the following: *A. vitis* >> *E. amylovora* > *P. syringae > X. juglandis.*	[17]
**34**	Sol−gel and electrospinning approaches in the presence of different amounts of air–argon mixtures	TiO_2_	50–300	Electrospun nanofibers	3000 µg/mL 6000 µg/mL	Gr+ Gr−	*MRSA* *P. aeruginosa*	TSB, 18 h, 30 °C	BC	TiO_2_ NFs were more operative against Gram-negative *P. aeruginosa* than Gram-positive *S. aureus*.	[159]
35	Microwave-assisted method	TiO_2_ Ba-TiO_2_	8–18 4–10	Sph	100 µg/mL >> >> >>	Gr+ Gr− Gr− Gr−	*B. subtilis* *V. cholera* *P. aeruginosa* *S. flexneri*	NA, 24 h, 37 °C	BS	The antibacterial activity was found to be higher for Ba-doped TiO_2_ nanoparticles compared to pure TiO_2_ NPs due to reduced particle size and high specific surface area, leading to enhanced particle surface reactivity to light and H_2_O adsorption.	[60]
36	Ultrasonic vibration of Ag-TiO_2_ compound nanoparticles (obtained by picosecond laser ablation in deionized water)	TiO_2_/Ag (TiO_2_ core, Ag shell)	~10–180 (average: 27)	Sph	20 μg/mL	Gr−	*E. coli*	Lysogeny broth (LB), 6 h, 37 °C + LB agar dish, 2 d, ~22 °C	BS	The antibacterial activity of the core-shell NPs was slightly better than that of the compound NPs at the same concentration under standard laboratory light conditions, and both were better than the TiO_2_ NPs but not as good as the Ag NPs.	[133]
37	Green synthesis of Ag-doped TiO_2_ nanoparticles using maple leaf extract	Ag/TiO_2_	45.90	Rods	4000 μg/mL >>	Gr+ Gr−	*S. aureus* *E. coli*	NA, 24 h, 37 °C	BS	The doping of Ag into TiO_2_ reveals the enhancement of inhibition growth against Gram-positive (*S. aureus*) and Gram-negative (*E. coli*).	[185]
38	A low-temperature sol−gel process using organic solvents (TO) and aqueous extract of mangrove leaves (TM) as media	TiO_2_	13.1 (TO) 8.3 (TM)	Amorph	5 μg/mL >> >>	Gr+ Gr+ Gr−	*S. aureus*, *E. faecalis*, *V. damsela*	NA, 12 h. (overnight), 37 ± 2 °C	BS	The green method utilising TM demonstrated resistance to all three bacteria types, while TO exhibited greater resistance, specifically against *V. damsela.* Notably, TM NPs exhibited inhibition comparable to Ciprofloxacin when used as a positive control.	[186]
**39**	Sol−gel method loaded with cardamom essential oil (CEO)	CEO@ TiO_2_	100–1000 (average: 335.6)	Capsule-like	18.75 μg/mL 25 μg/mL 18.75 μg/mL	Gr− Gr+ Gr−	*E. coli*, *B. subtilis*, *K. pneumoniae*	NB, 24 h, 37 °C	BC	The results demonstrate that CEO/TiO_2_ conjugates exhibit more potent antibacterial activity against all the tested bacteria than CEO or TiO_2_ nanoparticles alone.	[187]
40	Synthesis from TiOSO_4_ (NPs) and hydrothermal method (NWs)	TiO_2_	~80 ~100	Sph, Wire	20,000 μg/mL	Gr+	*S. aureus*	LB agar, 12 h. (overnight), 37 °C	BC	The anti-staphylococcal activity of TiO_2_ nanowires was better than the nanoparticles.	[188]
**41**	Green synthesis using an enzyme alpha-amylase	TiO_2_	30–70 (average: 50)	Sph	62.50 μg/mL >>	Gr+ Gr−	*S. aureus*, *E. coli*	NA, 18 h, 37 °C	BC	The morphology and shape depend upon the concentration of the alpha-amylase enzyme. The biosynthesized NPs show good bactericidal effects against both Gram-positive and Gram-negative bacteria.	[174]
**42**	Green synthesis using *Azadirachta indica* leaf extract	TiO_2_	25–87 (SEM)	Sph	20.83 μg/mL 16.66 μg/mL 25 μg/mL 10.42 μg/mL 10.42 μg/mL	Gr+ Gr− Gr+ Gr− Gr−	*S. aureus*, *K. pneumoniae*, *B. subtilis*, *S. typhi*, *E. coli*,	MHA, 24 h, 37 °C	BC	Green synthesis of TiO_2_ NPs was achieved because of the presence of terpenoids, flavonoids, and proteins in *Azadirachta indica,* as these bioactive compounds were responsible for the synthesis of these NPs.	[189]
**43**	Sol−gel method	TiO_2_	64.77 ± 0.14	Irregular sph	8–64 μg/mL (av: 46.90) <1 μg/mL	Gr− >>	*22 P. aeruginosa* Isolates (wound exudate, ear discharge) *3 P. aeruginosa* Isolates (urine specimen)	MHA, 18–24 h, 37 °C	BS	The combination of TiO_2_ NPs and cefepime was found to show synergistic activity against all tested isolates, followed by ceftriaxone (96%), amikacin (88%), and ciprofloxacin (80%).	[190]
44	Hydrothermal and solvothermal conditions.	TiO_2_	3~8	-	100 μg/mL >> >>	Gr+ Gr− Gr−	*MRSA*, *P. aeruginosa*, *E. coli*	LB agar, 12 h. (overnight), 37 °C	BS	The antibacterial activity against pathogens was as follows: MRSA > *E. coli* > *P. aeruginosa*. The synthesized TiO_2_ NPs were genotoxic/mutagenic.	[140]
**45**	Microwave-irradiation-assisted hybrid chemical approach	TiO_2_	28.3 ± 3.1	-	15 µg/mL	Gr−	*E. coli*	LB, 37 °C	BC	The one reason that can be assured for microbial cell death after treatment with nano-titania is reactive oxygen generation and an increase in membrane permeabilization, not superoxide generation.	[191]
**46**	Hydrothermal technique	TiO_2_ TiO_2_ TiO_2_ S-TiO_2_ S-TiO_2_ S-TiO_2_	25–32 >> >> 27–45 >> >>	Sph >> >> Sph >> >>	0.1 µg/mL 0.1 µg/mL 100 µg/mL 0.01 µg/mL 0.1 µg/mL 0.1 µg/mL	Gr− Gr− Gr− Gr− Gr− Gr−	*V. cholerae*, *E. coli*, *P. aeruginosa* *V. cholera* *E. coli*, *P. aeruginosa*	LB, 24 h, 37 °C	BS, BC	The 40% Sulfur (S) doped TiO_2_ sample showed the highest antibacterial activity against the *V. cholerae* by killing 71% bacteria at a minimum inhibitory concentration of 0.01 μg/mL. NPs are non-cytotoxic against hepatocellular carcinoma (Huh-7) human cell lines	[136]
**47**	Ultrasound method	TiO_2_	33.56	Amorph	9.7 µg/mL 19.5 µg/mL 19.0 µg/mL 9.7 µg/mL 19.5 µg/mL	Gr− Gr+ Gr− Fungus Gr+	*E. coli*, *S. aureus*, *P. aeruginosa*, *C. albicans*, *B. subtilis*	NA, 24 h, 37 °C	BS, FS	The TiO_2_ nanoparticles synthesized by ultrasound method can be good inorganic antimicrobial agents.	[192]
48	Hydrothermal synthesis	TiO_2_	70.80	Sph	50 µg/mL >> >>	Gr+ Gr− Gr−	*B. subtilis*, *K. pneumoniae*, *S. typhi*	MHA, 24 h, 37 °C	BS	At the maximum concentration tested (150 mg/L), TiO_2_ NPs showed strong inhibitory action against *B. subtilis* (zone of inhibition 8.4 mm), *K. pneumoniae* (zone of inhibition 8.8 mm), and *S. typhi* (zone of inhibition 9.3 mm).	[193]
49	Sol−gel method using diethanolamine, acetic acid, and propionic acid solvents	TiO_2_	5–25	Sph	250 mg/mL >> >> >>	Gr− Gr− Gr− Gr−	*ATCC. E. coli*, *MDR. E. coli*, *ATCC P. aeruginosa*, *MDR. P. aeruginosa*	MHA, 24 h, 37 °C	BS	The diethanolamine, acetic acid, and propionic acid solvents showed comparatively good antibacterial activity due to the rutile phase and pH of these solvents, which modify the properties of TiO_2_.	[194]
50	Sol–gel method	TiO_2_	68	Sph	50,000 µg/mL >>	Gr− Gr+	*E. coli*, *S. aureus*	MHA, 24 h, 37 °C	BS	MMT test showed no toxicity of TiO_2_ NPs; the antibacterial inhibitory effect of TiO_2_ NPs at 200 mg/mL concentrations exhibited superior antibacterial activity at 15.9 ± 0.1 and 14.0 ± 0.1 against *S. aureus* and *E. coli*, respectively. In conclusion, colloidal solutions with high stability were successfully synthesized, contributing to decreased dimensions and increased antibacterial properties.	[195]
**51**	Green synthesis using *Iranian propolis* extracts	TiO_2_	21	Quasi-sph	10 mg/mL 1.25 mg/mL 5 mg/mL 2.5 mg/mL 5 mg/mL 5 mg/mL	Fungus Gr+ Gr+ Gr+ Gr+ Gr+	*C. albicans*, *S. sobrinus*, *S. mutans*, *S. sanguinis*, *S. salivarius*, *L. acidophilus*	TSB (bacteria) or YPD (fungus), 24 h, 37 °C	BC	There were no significant cytotoxicity effects. NPs from propolis extracts have less toxic effects and are user-friendly, eco-friendly, and economical materials. Pro1TiO_2_ (Khalkhal sample) NPs may be considered the best candidate for clinical application.	[196]
**52**	Green synthesis from *Juniperus phoenicea* (L.) leaf extract	TiO_2_	10–30	Sph	40 µg/mL 40 µg/mL 80 µg/mL 80 µg/mL 40 µg/mL 20 µg/mL 40 µg/mL	Gr+ Gr+ Gr− Gr− Fungus Fungus Fungus	*S. aureus*, *B. subtilis*, *E. coli*, *K. pneumoniae*, *S. cerevisiae*, *Asp. niger*, *Pen. digitatum*	SDA (fungus) and MHA (bacteria), 24 h, 37 °C	BS, FS	Some components such as elemol, linalool, and hydrocinnamic acid in *Juniperus phoenicea* plant due to having hydroxyl functional groups act as reducing agents.	[197]
**53**	Synthesis NPs by sonication with *Ganoderma lucidum* extract	TiO_2_ + Ganoderma extract	-	-	156.2 µg/mL 78.12 µg/mL	Gr− Gr+	*P. aeruginosa*, *MRSA*	TSB, 24 h, 37 °C	BS	Collectively, the combination of TiO_2_ NPs and *Ganoderma* extract was more able to reduce viable cells, especially against MRSA isolate, and had almost the same effect as vancomycin.	[198]
54	Synthesis by plant extracts	TiO_2_	12	hexagonal	25 µg/mL >> >> >> >> >> >> >> >> >> >>	Gr− Gr− Gr− Gr− Gr− Gr+ Fungus Fungus Fungus Fungus Fungus	*E. coli*, *P. mirabilis*, *V. cholerae*, *P. aeruginosa*, *S. typhimurium*, *S. aureus*, *A. nidulans*, *A. fumigatus*, *A. niger*, *A. terreus*, *A. flavu*	MHA, 48 h, 37 °C (bacteria), PDA, 5 d, 28 °C (fungus)	BS FS	TiO_2_ NPs have concentration-dependent antibacterial activity against bacterial pathogens such as *E. coli*, *P. mirabilis*, *V. cholerae*, *P. aeruginosa*, *S. typhimurium*, and *S. aureus* at 100 μg/mL concentration. Furthermore, these TiO_2_ NPs showed remarkable antifungal activity against aspergillosis-causing fungal pathogens such as *A. niger*, *A. fumigatus*, *A. nidulans*, and *A. flavus* at 100 μg/mL.	[199]
55	Green synthesis mediated by extract of clove and ginger herbal formulation	TiO_2_	-	-	25 µL	Gr+	*Lactobacillus*	MHA, 4 h, 37 °C	BS	The results show that as the concentration increases, the value of optical density decreases, which proves that a bactericidal process occurs that results in a reduced bacterial count.	[200]
**56**	The metabolic doping of cultured diatom cells with titanium, pyrolysis of the doped biomass, and chemical doping of silver	AgNPs/TiO_2_/DBP	10–20	Quasi-sph	2.5 mg/mL 1.25 mg/mL 2.5 mg/mL	Gr+ Gr− Gr−	*S. aureus*, *K. pneumonia*, *E. coli*	-	BS	Remarkably high antibacterial activity was observed for the synthesized nanocomposites against Gram-positive *S. aureus* and Gram-negative *K. pneumoniae* and *E. coli* strains, both laboratory-cultivated and clinical isolates. DPB—diatom pyrolysed biomass	[161]
57	Hydrothermal-assisted synthesis using a novel β-galactosidase isolated from the seed extract of *Melilots indica*	β-gal-TiO_2_	27	Sph	10 µg/mL 5 µg/mL	Gr− Gr+	*E. coli*, *S. aureus*	NA, 24 h, 37 °C	BS	The decrease in inhibitory efficacy at increased β-gal-TiO_2_ NPs concentrations could be attributed to the aggregation of some particles, thereby increasing the particle size and reducing the accessible surface area for nanoparticles.	[201]
58	Co-precipitation method using titanium tetra isopropoxide and hydrochloric acid as a precursor	α-TiO_2_ (Rutile)	16.65	Cuboid	10 g/disc	Gr− Gr− Gr+	*K. pneumoniae*, *E. coli*, *S. aureus*	MHA, 24 h, 37 °C	BS	TiO_2_-based NPs have highly developed surface chemistry, chemical stability, and a smaller size than a microorganism, making it easier for them to interact with the microorganisms.	[61]
59	Bio-modification of TiO_2_ nanoparticles surface	Tryptophan-TiO_2_	5–27	Amorph	45.7 µg/mL (EC_50)	Parasite	*T. gondii*	DMEM, 24 h, 37 °C	BS	Tryptophan-TiO_2_ nanoparticles (NPs) show selective anti-parasitic activity. Surface modification with amino acids, such as l-tryptophan not only enhanced the anti-parasitic action of TiO_2_ but also improved the host biocompatibility.	[202]
60	Green synthesis from leaf extracts of two plant species (*Trianthema portulacastrum*, *Chenopodium quinoa*)	TiO_2_	6–8	Granule	25 μL/mL	Fungus	*U. Tritici*	PDA, 5 days., 25 ± 2 °C	FS	The calcinated TiO_2_ NPs exhibited substantial antifungal activity against *U. tritici* compared to non-calcinated NPS. All of the NPs produced using different procedures (also used the sol−gel method for comparison) and at different doses showed considerable antifungal activity against *U. tritici*.	[203]
**61**	Biosynthesis using *Aloe vera* L. aqueous leaf extract	TiO_2_ + *T.* cf. *asperellum* extract	10–25	Tetragonal	25 µg/mL	Fungus	*B. sorokiniana*	PDA, 5 d, 28 °C	BS, BC	Green synthesized TiO_2_ NPs positively increased the host plant’s tolerance against this disease by inducing osmolytes and antioxidant defense-related enzyme production.	[204]
62	Commercial from US Research Nanomaterials	TiO_2_	30	-	200 µg/mL >> >> >>	Gr+ Gr+ Gr+ Gr+	*L. reuteri*, *L. gasseri*, *B. animalis*, *B. longum*	MRS, 24 h, 37 °C	BS	The inhibitory effects of TiO_2_ NPs were associated with cell membrane damage. TiO_2_ NPs caused alterations in multiple metabolic pathways of gut bacteria.	[205]
63	Synthesis using *Beta vulgaris* (beetroot) extract	TiO_2_/*Beta vulgaris* extract	12	Sph	1000 µg/mL 500 µg/mL Nil Nil	Gr− Gr− Gr+ Gr+	*E. coli*, *P. aeruginosa*, *S. aureus*, *S. mutans*	MHA, 24 h, 37 °C	BS	Antibacterial assays reveal moderate activity against Gram-negative bacterial strains, while no activity is observed against Gram-positive bacterial strains.	[206]
64	Biosynthesis using probiotic *Bifidobacterium bifidum*	TiO_2_/*B. bifidum* components	81	Oval	16 mg/mL 16 mg/mL 16 mg/mL 32 mg/mL 32 mg/mL	Gr− Gr− Gr− Gr− Gr−	*P. aeruginosa*, *A. baumani*, *K. pneumonia*, *E. coli*, *S. typhi*	NA, 24 h, 37 °C	BS	*Bifidobacteria* were found to engage in huge inhibition activity against Gram-negative bacteria, i.e., intestinal Salmonella serovar Typhimurium SL1344 and *Escherichia coli* C1845. The inhibition mechanism was examined and found to be dependent on lowering the pH in the medium and producing organic acids, especially acetic acid and lactic acid.	[207]
**65**	Hydrothermal route using novel biogenic source *Piper betel* leaf extract and chemogenic source nitric acid as capping and reducing agents	TiO_2_/*Piper betel* extract	8 75	Sph	25 µg/mL 50 µg/mL	Gr+ Gr−	*S. aureus*, *E. coli*	NA, 24 h, 37 °C	BS	Biogenic synthesized NPs act as more effective antibacterial agents than chemogenic-synthesized NPs.	[208]
**66**	Modification of commercial PAMAM and TiO_2_ to novel nanocomposite	PAMAM/TiO_2_	50	Sph	4 µg/mL 2 µg/mL	Gr+ Gr−	*S. aureus*, *E. coli*	NB, 24 h, 37 °C	BC	FE-SEM analysis revealed morphological variations and the mechanism of killing and trapping the bacteria by nanocomposite. In the MIC and MBC values range, the cytotoxicity effect of nanocomposite on the AGS cell line was relatively lower. PAMAM—poly-amidoamine dendrimer macromolecule	[209]
67	Green synthesis method using Orange peel extract (*Citrus sinensis*) and chemical method	TiO_2_/*Citrus sinensi* extract	21.61 17.30	Porous angular	6.75 mg/mL >> >>	Gr+ Gr− Gr−	*S. aureus*, *E. coli*, *P. aeruginosa*	NB, 24 h, 37 °C	BS	TiO_2_ nanoparticles prepared using the biological method exhibit good results compared to nanoparticles prepared by the chemical method.	[72]
68	Hydrothermal method	CQD-TiO_2_ BC/CQD-TiO_2_	22.23	Porous fibers	Nil 0.5 µg/mL	Gr− Gr+	*E. coli*, *S. aureus*	Netrin agar, 24 h, 37 °C	BS	The antibacterial activity against *E. coli* was much lower than when it was against *S. aureus*. This concept is because of the discrepancy in the structure of the cell walls between Gram-negative and Gram-positive bacteria.	[68]
**69**	Synthesis porous TiO_2_ NPs and PVA-PEG/TiO_2_ composites using the sol−gel technique	PVA-PEG/TiO_2_	15	Sph	100 µL	Gr−	*E. coli*	LB, 24 h, 37 °C	BS	Antibacterial activity experiments, TiO_2_ 19.9%, PVA/TiO_2_ 24.4%, and PEG/TiO_2_ 26.2% eliminated *E. coli* bacteria.	[210]
70	Microwave-assisted green method using *Andrographis paniculata* as fuel	TiO_2_/*Andrographis paniculata* extract	25	Sph	41 µg/mL 36 µg/mL 35 µg/mL 42 µg/mL 30 µg/mL	Gr− Gr− Gr− Gr+ Gr−	*E. coli*, *S. flexneri*, *P. aeruginosa*, *S. aureus*, *K. pneumoniae*	NB, 24 h, 37 °C	BS	NPs TiO_2_ NPs led to mechanical damage of cell membrane and significant cell division inhibition compared to standard antibiotics (Streptomycin).	[211]
**71**	Sol−gel technique using titanium tetra isopropoxide (TTIP) as a precursor	TiO_2_ Ag-TiO_2_	15 16	Sph	1 µg/mL >> >>	Gr− Gr− Gr+	*E. coli*, *P. aeruginosa*, *S. aureus*	NA, 12 h, 37 °C	BS	Significant increases in the sizes of zones of inhibition with the increase in silver capping (3–7%) as compared to pure TiO_2_-NPs were observed against Gram-negative and Gram-positive bacteria.	[212]
72	One-pot and one-step green synthesis using Grape seed extract (GSE) proanthocyanin (PAC) polyphenols	Grape seed extract/TiO_2_	18.42 ± 1.3	Sph	1.56 µg/mL 0.78 µg/mL	Gr− Gr+	*P. aeruginosa*, *S. saprophyticus*	BHI, 24 h, 37 °C	BC	PACs on the GSE-TiO_2_-NPs surface significantly enhanced the antibacterial activities in terms of confinement of the biofilm formation, plausibly through the entrapment of AL-2 QS signal molecules, attenuating the bio-actives (e.g., proteins, enzymes, nucleic acids, and EPS), biofilm matrix, increasing cellular uptake and ROS mediated robust oxidative stress.	[162]
**73**	Biosynthesis using titanium tetrabutoxide as a precursor in the presence of *Kniphofia foliosa* root extract within different ratios	TiO_2_/*Kniphofia foliosa* extract	~9	Sph	35 mg/mL >> >> >>	Gr+ Gr+ Gr− Gr−	*S. aureus*, *S. pyogenes*, *E. coli*, *K. pneumonia*	MHA, 24 h, 37 °C	BS	Among the different ratios, TiO_2_ (1:1) NP shows better performance towards Gram-negative bacteria due to its smaller average crystalline size and uniform morphology than the other two ratios of TiO_2_ NPs. The antibacterial activity of the ethanolic root extract of *Kniphofia foliosa* itself showed better performance towards Gram-negative bacteria than NPs of TiO_2,_ which might be due to the antibacterial activity of the residue of ethanol left with the plant extract.	[63]
74	Green synthesis using aqueous extract of *Acacia nilotica* as bio-reductant	Ag-TiO_2_/*Acacia nilotica* extract	11.25	Sph	64 µg/mL 64 µg/mL 128 µg/mL 64 µg/mL	Gr− Gr+ Gr− Yeast	*E. coli*, *S. aureus*, *P. aeruginosa*, *C. albicans*	MHA, 24 h, 37 °C	BS	The order of antimicrobial activity was found to be *E. coli* > *C. albicans* > *MRSA* > *P. aeruginosa.*	[213]
**75**	Green synthesis using aqueous leaf extract of *Coleus aromaticus*	TiO_2_/*Coleus aromaticus* extract	12–33	Sph	15 µg/mL >> >> >> >> >>	Gr− Gr− Gr+ Gr− Gr+ Gr+	*S. boydii*, *V. cholerae*, *B. cereus*, *A. hydrophila*, *E. faecalis*, *B. megaterium*	MHA, 24 h, 37 °C	BS	The synthesized TiO_2_ NPs had an excellent antibacterial potential against *E. faecalis* (33 mm), followed by *S. boydii* (30 mm)	[214]
76	Green synthesis using *Cymodocea serrulate* aqueous extract	TiO_2_/*Cymodocea serrulate* extract	55–117	Sph	180 μg/mL 160 μg/mL	Gr+ Gr−	*MRSA*, *V. cholerae*	MHA, 24 h, 37 °C	BS	The TiO_2_ NPs treated results were exhibited maximum antibacterial activity against MRSA and *V. cholerae* comparatively.	[215]
**77**	Green synthesis mediated *Spirulina*	TiO_2_/*Spirulina* components	55 ± 15	Sph	3.906 µg/mL 15.625 µg/mL 15.625 µg/mL 31.25 µg/mL	Gr+ Gr− Gr+ Gr−	*S. aureus*, *P. aeruginosa*, *E. faecalis*, *E. coli*	MHA, 24 h, 37 °C	BS	The nanoparticles exhibited significant inhibitory zones of 22 ± 3, 17 ± 4, 11 ± 2, and 15 ± 3 nm at 80 μg/mL against MRSA, *P. aeruginosa*, *E. coli*, and *E. faecalis*, respectively.	[216]
**78**	Green synthesis using leaf extract of *Mentha arvensis*	TiO_2_/*Mentha arvensis* extracts	20–70	Sph	10 mg/mL Nil Nil 10 mg/mL Nil Nil	Gr− Gr+ Gr− Fungus Fungus Fungus	*P. vulgaris*, *S. aureus*, *E. coli*, *A. niger*, *A. cuboid*, *A. fumigates*	NA, 24 h, 37 °C	BS	Synthesized TiO_2_ nanoparticles show maximum zone of inhibition against the *Proteus vulgaris* bacteria (at 30 mg/mL) and show significant antifungal activity against *Aspergillus niger*.	[217]
79	Green synthesis using *Psidium guajava* extract	TiO_2_/*Psidium guajava* extract	32.58	Sph	20 µg/mL >> >> >> >>	Gr+ Gr− Gr− Gr− Gr−	*S. aureus*, *E. coli*, *A. hydrophila*, *P. mirabilis*, *P. aeruginosa*	MHA, 24 h, 37 °C	BS	The synthesized TiO_2_ NPs showed more antibacterial activity than the standard tetracycline antibiotic	[218]
80	Green synthesis using *Acorus calamus* leaf extract	TiO_2_/*Acorus calamus* extract	15–40	Globular	10 µg/mL >> >> >>	Gr− Gr− Gr+ Gr+	*E. coli*, *P. aeruginosa*, *B. subtilis*, *S. aureus*	MHA, 24 h, 37 °C	BS	Biosynthesized TiO_2_ showed excellent antimicrobial activity against the selected Gram-positive over Gram-negative pathogenic bacteria in comparison to bare TiO_2_. NPs disrupt the outer cell of bacteria, which is primarily responsible for bacterial death.	[219]
81	Green Synthesis via *Eucalyptus globulus* L. Extract	Ag-TiO_2_/*Eucalyptus globulus* extract	11–14	Sph	13.33 μg/μL	Gr+ Gr−	*S. aureus*, *E. coli*	LB, 24 h, 37 °C	BS	The effect of NPs is more significant for Gram-negative bacteria because of their thinner cell wall, compared to 30 nm for Gram-positive bacteria.	[220]
82	Green synthesis using aqueous extract of *W. somnifera*	TiO_2_/*W. somnifera* extract	50–90	Sph, Square	64 µg/mL 8 µg/mL 32 µg/mL 32 µg/mL 32 µg/mL 64 µg/mL	Gr+ Gr− Gr− Gr− Gr+ Yeast	*L. monocytogenes*, *S. marcescens*, *E. coli*, *P. aeruginosa*, *MRSA*, *C. albicans*	NB, 24 h, 37 °C	BC	Among bacteria, the highest inhibition of 71% was recorded in MRSA, and the lowest was recorded in L. monocytogenes (43%).	[221]
**83**	Green synthesis using *Aloe barbadensis* mill	TiO_2_/*Aloe barbadensis* extract	20	Sph	31.25 µg/mL	Gr−	*P. aeruginosa*	MHA, 24 h, 37 °C	BC	A noticeable suppression in the cell viability by 30.76 ± 3.96% of *P. aeruginosa* in the biofilm mode was found in the presence of TiO_2_ NPs	[222]
**84**	Bacterial-mediated *Bacillus subtilis* MTCC 8322 using TiCl_4_ as a precursor	TiO_2_/*Bacillus subtilis* components	80–120	Sph	5 µg/mL 8 µg/mL	Gr+ Gr−	*B. subtilis*, *E. coli*	NA	BS	The TiO_2_ NPs exhibited antibacterial activity against *Bacillus subtilis* MTCC 8322 at a lower dose, while against *E. coli* 8933, only a higher dose exhibited antibacterial activity.	[64]
**85**	Bacterial-mediated *Staphylococcus aureus*	TiO_2_/*Staphylococcus aureus* components	20	Sph	10 mg/mL >>	Gr− Gr+	*E. coli*, *B. subtilis*	NA, 12 h. (overnight), 37 °C	BS	The differential sensitivity of Gram-negative and Gram-positive bacteria towards nanoparticles may depend upon their cell outer layer attribute and their interaction with the charged TiO_2_ nanoparticles. It was observed that Gram-negative bacteria are more sensitive than Gram-positive bacteria.	[70]
86	Green synthesis using *Caesalpinia pulcherrima* flower extract, *Nervilia aragoana* leaf extract, and *Manihot esculenta* peel extract	TiO_2_*/C. pulcherrima*, *N. aragoana*, *M. esculenta* plants extracts	15–28	Sph	50 µg/mL >> >> >>	Gr+ Gr− Gr− Yeast	*S. aureus*, *E. coli*, *P. aeruginosa*, *C. albicans*	MHA, 12 h.	BS	The obtained results showed that the TiO_2_ sample revealed better antibacterial activity in *S. aureus* than in the *E. coli* bacterial strain.	[223]
87	Synthesis using *Planomicrobium* sp.	TiO_2_/*Planomicrobium* components	>8.89	Amorph	0.1 µg/mL >>	Gr+ Gr−	*B. subtilis*, *K. Planticola*	MHA, 24 h, 37 °C	BS	The differential sensitivity of Gram-negative and Gram-positive bacteria towards NPs may depend upon their cell outer layer attribute and their interaction with the charged TiO_2_ NPs.	[224]
88	Green synthesis using Edible Mushroom (*Pleurotus djamor*) Extract	TiO_2_/*Pleurotus djamor*) extract	31	Sph	5 mg/mL >> >> >> >> >> >>	Gr+ Gr+ Gr+ Gr− Gr+ Gr− Gr−	*B. cereus*, *B. subtilis MDB*, *C. diphtheriae*, *E. coli*, *S. aureus*, *P. fluorescens*, *Serratia sp.*	MHA, 24 h, 37 °C	BS	The highest rate of inhibition zone was recorded in *P. fluorescens* (33 ± 0.2 mm), *S. aureus* (32 ± 0.4 mm), and *C. diphtheriae* (32 ± 0.1 mm) followed by others.	[69]
89	Commercial Degussa-P25 TiO_2_	TiO_2_	25	-	350 μg/mL	Gr−	*P. aeruginosa*	MHA, 24 h, 37 °C	BS	Exposure to UV irradiation of 60 min has been shown to greatly enhance the antibacterial efficacy of TiO_2_ nanoparticles against MDR *P. aeruginosa*.	[225]
90	Biosynthesis-mediated *Avicennia marina*	TiO_2_/*Avicennia marina* extract	30	Sph	100 μg/mL >>	Gr− Gr+	*E. coli*, *S. aureus*	LB, 24 h, 37 °C	BS	It was found that the bactericidal effect of the biosynthesized TiO_2_ increased with increasing concentrations from 100 to 300 μg/mL; thereafter, a decrease was observed, and again, the bactericidal effect was increased from 400 to 1000 μg/mL. This trend was observed for both *E. coli* and *S. aureus*.	[66]
91	Green synthesis using *Artemisia haussknechtii* leaf extract	TiO_2_/*Artemisia haussknechtii* extract	92.58 ± 56.98	Sph	40 μg/mL 20 μg/mL 4 μg/mL 4 μg/mL	Gr− Gr+ Gr+ Gr−	*E. coli*, *S. aureus*, *S. epidermidis*, *S. marcescens*	MHA, 24 h, 37 °C	BC	TiO_2_ NPs had no significant effect on *E. coli* ATCC 25,922 and *S. aureus* ATCC 43300, but there was antibacterial impact on *S epidermidis* ATCC 12258, *S. marcescens* ATTC13880 as lack of growth. The results of the synthesis and antibacterial properties of silver and copper nanoparticles were also presented in the study.	[65]
**92**	Green synthesis using *Limon citrus* extract	TiO_2_/*Limon citrus* extract	200 *	Sph	12.5 µg/mL 12.5 µg/mL 18.75 µg/mL 12.5 µg/mL	Gr− Gr− Gr+ Gr+	*E. coli*, *Klebsiella* sp., *MRSA*, *Bacillus*	NB, 24 h, 37 °C		The most accepted mechanism for antibacterial activity is based on the generation of reactive oxygen species associated with the photocatalytic activity of TiO_2_ nanostructures. Also, the antibacterial efficiency of the green-prepared TiO_2_ NPs was compared with NPs prepared via a chemical process.	[226]
**93**	Sol−gel method	TiO_2_	22.41	Sph	65 mg/mL 200 mg/mL 100 mg/mL 144 mg/mL 72 mg/mL 100 mg/mL 100 mg/mL 20 mg/mL	Gr+ Gr+ Gr+ Gr+ Gr− Gr− Gr− Gr−	*S. fecalis* *S. pyogenes* *S. saprophyticus* *S. epidermidis* *E.coli MDR* *E.coli* *A. hydrophila* *S. dysenteriae*	MHA, 24–48 h, 37 ± 2 °C	BS	Titanium dioxide (TiO_2_) nanoparticles demonstrated antifungal and antibacterial activities.	[227]
**94**	Hydrothermal method	Commercial TiO_2_	8–10 (*) 90–100 (*)	-	10 µg/mL (*) 50 µg/mL (**) 10 µg/mL (*) 50 µg/mL (**)	Gr− Gr+	*E. coli NCIM 2065* *S. aureus ATCC 6538*	NB, 12 h. (overnight), 37 °C	BC	TiO_2_ NPs 8–10 nm have profound action on *E. coli,* while *S. aureus* was not affected. TiO_2_ NPs 90–100 nm have very little effect on both organisms.	[142]
**95**	Hydrothermal method	TiO_2_	67.60	Rods	240 µg/mL	Gr−	*R. solanacearum*	-	BS	Genomic DNA injury might be due to the intracellular production of reactive oxygen species (O_2_, O^2−^ and OH) which was stimulated by TiO_2_ NPs.	[228]

**Notes**: The article numbers in **bold** include a test to determine the minimum inhibitory concentration (MIC); the remaining articles present the lowest concentrations used in studies on antibacterial activity. ^1^—here and below, quantitative data in dimensions different from µg/mL are taken from the original works. “-”—a characteristic that was not unspecified by the authors. >>—the value repeats the previous one. *—size determined by the image provided by the authors. **—concentration at which the maximum bactericidal effect was observed. Nil—no result. Gr−—Gram-negative bacteria. Gr+—Gram-positive bacteria.

## 8. Conclusions

The growth of antibiotic resistance is one of the most dangerous challenges to global healthcare. Metal oxide NPs, and TiO_2_ in particular, look like attractive candidates for the role of new antimicrobial agents. TiO_2_ NPs are already used in many areas of technology, medicine, and agriculture. In addition, TiO_2_ NPs are biologically inert and have low toxicity to humans and animals. All this makes TiO_2_ NPs attractive as antimicrobial agents. The mechanisms of antimicrobial action of TiO_2_ NPs include contact, photocatalytic, and ROS-mediated action. Antibacterial and antifungal properties of TiO_2_ NPs weakly depend on the type of microorganism against which they are used. Therefore, TiO_2_ NPs can be considered as a universal antimicrobial agent of a broad spectrum of action. The dependence of antimicrobial properties on their size is somewhat more pronounced: the dependence has a complex form. The shape, method of synthesis, and modification of the composition of TiO_2_ NPs have a more pronounced effect on their resulting antibacterial and antifungal activity. The greatest antimicrobial potential is possessed by amorphous NPs with a size of 20–60 nm, modified with metals or components of plant extracts and/or bacterial nature compounds. The patterns shown by us can be useful in the development of new methods and approaches for the synthesis of TiO_2_ NPs with improved antimicrobial activity. We believe that further ways to improve the antimicrobial properties of TiO_2_ NPs lie in the development of new and improvement of known methods of surface modification of TiO_2_ NPs with metals and/or plant extracts components. Methods for synthesizing amorphous NPs also look promising. However, the strategies for increasing the antimicrobial activity of TiO_2_ NPs are not exhausted by the above. New approaches to the analysis of the dependence of the antimicrobial activity value on the characteristics of NPs are needed to search for new factors to increase antibacterial activity.

## Data Availability

The raw data supporting the conclusions of this article will be made available by the authors without undue reservation.

## Abbreviations

AV	Antivirus
BC	Bacterial cellulose
BC	bactericidal effect
BHI	Brain heart infusion broth
BS	bacteriostatic effect
CQD	Carbon Quantum Dots;
DMEM	Dulbecco’s Modified Eagle Medium
DMEM	Modified Eagle Medium
LB	Luria brother
MDR	multidrug-resistant
MHA	Mueller-Hinton agar
MHB	Mueller-Hinton Brotherhood
MRSA	Methicillin-resistant Staphylococcus aureus
NA	Nutrient agar
NB	Nutrient broth
PAMAM	poly-amidoamine dendrimer macromolecule
PDA	Potato Dextrose Agar
PEG	polyethylene glycol
PVA	Polyvinyl alcohol
SDA	Sabouraud dextrose agar
TSB	Trypticase soy broth
YNB	Yeast nitrogen base
YPD	Yeast peptone dextrose broth
